# Efficacy evaluation of photodynamic therapy for oral lichen planus: a systematic review and meta-analysis

**DOI:** 10.1186/s12903-020-01260-x

**Published:** 2020-11-04

**Authors:** Yuqing He, Jiaxin Deng, Yi Zhao, Huiqian Tao, Hongxia Dan, Hao Xu, Qianming Chen

**Affiliations:** State Key Laboratory of Oral Diseases, National Clinical Research Center for Oral Diseases, Chinese Academy of Medical Sciences Research Unit of Oral Carcinogenesis and Management, West China Hospital of Stomatology, Sichuan University, No. 14, Sec.3, Ren Min Nan Road, Chengdu, 610041 Sichuan China

**Keywords:** Photodynamic therapy, Oral lichen planus, Efficacy evaluation, Meta-analysis

## Abstract

**Background:**

Photodynamic therapy (PDT) is a new option for oral lichen planus (OLP) management; however, there are different opinions on the efficacy of PDT for OLP. The aim of this study was to comprehensively assess the efficacy of PDT in the treatment of OLP and compare PDT with steroid therapy.

**Methods:**

A systematic review and meta-analysis were conducted to assess the curative effect of PDT. Five electronic databases were searched, PubMed, Web of Science, the Cochrane Library, Embase, and EBSCO up to 1 December, 2019. Random and fixed effects models for pooled estimates calculation were used and the Meta package of R was applied.

**Results:**

Pooled estimates revealed that, after PDT, the lesion size decreased by 1.53 cm^2^ (95% confidence interval (CI): 0.71–2.35) after PDT and the partial response (PR) was 0.77 (95% CI: 0.65–0.85). The visual analogue scale (VAS) score decreased by 3.82 (95% CI: 2.80–4.85) and the Thongprasom sign score decreased by 1.33 (95% CI: 0.56–2.10) after PDT. Subgroup analyses revealed that the 5-aminolevulinic acid (5-ALA) was more effective than methylene blue (MB), with a PR of 0.87 (95% CI: 0.80–0.91). The topical use of 5-ALA yielded a better response than gargling methylene blue. In terms of VAS, the diode laser showed a better clinical PR in the treatment of OLP. In terms of changes in lesion size, the efficacy of the semiconductor laser was higher than that of the diode laser. PDT had a similar efficacy to topical corticosteroids, as shown by pooled estimates of five randomised controlled trials with 139 lesions.

**Conclusion:**

This systematic review indicates that PDT is an effective treatment modality for the management of OLP. PDT is as effective as topical corticosteroid in the treatment of OLP and could be used for cases resistant to steroids or when steroids are contraindicated.

## Background

Oral lichen planus (OLP), a chronic immune-mediated, inflammatory, and psychosomatic condition that frequently affects the oral mucosa in a typical bilateral pattern, often presents as pain and a burning sensation [1]. OLP has an overall prevalence of about 2.2% [2]. The most common is the reticular type, which has a white lacy appearance. Other forms include erosive, atrophic, bullous, papular, and plaque-like. OLP is an oral potentially malignant disorder (OPMD) and has been linked to oral squamous cell carcinoma with a malignant transformation rate of 1.4% [3].

The aim of OLP management is to reduce the occurrence of symptoms and manifestation of lesions. Currently, the most common treatment for OLP is pharmacological therapy. Others include surgery, photodynamic therapy, and laser therapy. There is a large difference in the curative effect of the current treatments. In pharmacologic therapy, topical corticosteroids are usually prescribed, such as triamcinolone acetonide and dexamethasone [4]. However, long-term treatment with topical corticosteroids may cause obvious side effects, such as local pigmentation, oral candidiasis, and dry mouth [5]. Additionally, some studies have claimed that patients do not respond to drug treatment and the erosion does not heal, which increases the risk of canceration [6].

Photodynamic therapy (PDT) is a therapeutic method based on the photochemical and photobiological effects that are mediated by a photosensitiser (PS), which leads to cell damage at the lesioned tissue [7]. It is a minimally invasive treatment because it has the advantage of high selectivity. Thus, PDT causes only mild trauma and adverse reactions and is a new option for the treatment of OLP.

Currently, there are different opinions on the efficacy of PDT for OLP. One study has revealed that PDT has some effect in the symptomatic treatment of OLP in adult patients [8]. However, the authors used a small number of articles and did not perform subgroup analyses. On the contrary, according to a systematic review [9], PDT fails to exert any significant effect on the symptoms of OLP. A meta-analysis that reviewed 22 publications has shown that the partial response (PR) rate of OLP lesions to PDT is approximately 70%; however, this study analysed the effect of PDT on OPMD. Only six articles focused on OLP and the authors did not investigate the effect of different factors on the efficacy of PDT in OLP versus that in all OPMDs in the subgroup analysis [10].

These three reviews used a small number of articles focused on OLP and did not analyse the influence of factors, including the site of OLP lesion in mouth, type of PS, and administration method, that may be related to the final therapeutic response. Therefore, the aim of this systematic review and meta-analysis was to assess the efficacy of PDT in the treatment of OLP and compare the efficacy of PDT with steroid therapy. The results of this study will provide clinicians with a comprehensive understanding of the efficacy of PDT in OLP.

## Methods

### Study identification and selection criteria

The systematic review and meta-analysis were performed in accordance with the PRISMA statement [11], as detailed in Additional file 1: Table S1. This study has been registered on the International Prospective Register of Systematic Reviews (PROSPERO) under the registration number CRD42020160512.

Electronic and manual literature searches were conducted in the following five electronic databases: PubMed, Web of Science, the Cochrane Library, Embase, and EBSCO up to 1 December, 2019. Search terms were: “Photodynamic therapy” OR “PDT” AND “lichen planus” OR “oral lichen planus” OR “OLP.”

The inclusion criteria were: (a) original articles, clinical studies, and case series; (b) aim of the intervention was to evaluate the efficacy of PDT in the management of OLP; (c) lesion response was assessed and recorded; (d) articles published only in the English language; (e) clinical or histopathological diagnosis of OLP. The PICO questions below were applied:

Population (P): patients were diagnosed as OLP;

Intervention (I): patients were treated with PDT;

Comparison (C): condition of patients before PDT or topical corticosteroids;

Outcome (O): lesion response and lesion size of patients with OLP.

The exclusion criteria were: (a) reviews, abstracts, commentaries, letters to the editor, opinion articles, and animal studies; (b) inconsistent efficacy evaluation standard such that subsequent analysis cannot be performed; (c) individuals with idiopathic plaque-like lichen planus (non-erosive), lichenoid drug eruptions, or evidence of dysplasia in the tissue.

### Data extraction

Two authors (Z.Y. and D.J.X.) independently searched these five databases and assessed the titles and abstracts of all eligible publications. Details, including first author’s name, publication year, type of PS, disease types, method of administration, disease location, and number of lesions, were collected from the included studies. Four outcome measures were collected for the efficacy evaluation: (a) lesion response, including complete response (CR), which means lack of visible lesion confirmed by clinical evaluation, and PR, which means lesion size decreased by at least 20%; (b) changes in lesion size/area; (c) Thongprasom sign (TH): score of 0 for normal healthy mucosa, 1 for lesions with only white striae, 2 for mixed keratotic and atrophic or erythematous lesions smaller than 1 cm^2^, 3 for keratotic and atrophic or erythematous lesions larger than 1 cm^2^, 4 for erosive/ulcerative lesions smaller than 1 cm^2^, and 5 for erosive/ulcerative lesions larger than 1 cm^2^; (d) visual analogue scale (VAS) rated by participants (score: 0–10): 0 means no symptoms and 10 means severe symptoms, as perceived by the patient.

Other parameters used for qualitative synthesis included wavelength, energy density of the laser, duration of irradiation, lesion dressing, treatment interval, relapse during follow-up, and adverse reactions during and after PDT.

### Quality assessment

The included randomised controlled trials (RCTs) were assessed by the Cochrane Collaboration’s risk of bias assessment tool, with seven fields: random sequence generation, allocation concealment, blinding of participants and personnel, blinding of outcome assessment, incomplete outcome data, selective reporting, and other bias [12]. The included non-RCTs were assessed by the Downs-Black Checklist, with 29 items [13]. The quality assessment was independently performed by two authors (H.Y.Q. and D.J.X.). Any conflicts were fully discussed and the corresponding authors (X.H. and C.Q.M.) would make the final decision.

### Statistical analysis

The *I*^*2*^ statistic and heterogeneity statistic *Q* were calculated to assess heterogeneity and the random effect model was utilised to assess heterogeneity when the *I*^*2*^ statistic was more than 50% or the *p* value of the *Q* test was less than 0.05.

The outcome measures in this study were VAS, TH, size, and response (PR and CR). The weighted mean differences of the first three continuous indexes were pooled by the inverse variance method (for the fixed effects model) and restricted maximum-likelihood (for the random effects model). The proportion and odds ratio (OR) of the response were pooled by the inverse variance method (for the fixed effects model) and the DerSimonian-Laird method (for the random effects model).

Publication bias was evaluated using a funnel plot and weighted linear regression was used to test funnel plot asymmetry if the number of the studies was not less than 10. Publication bias could be ignored when the *p* value was greater than 0.05.

Sensitivity analysis was utilised in subgroup and influence analyses. The light source, type of PS, administration method, and lesion location were considered for subgroup analysis and the *u* test was applied for the differential test between different subgroups. The influence analysis of the pooled estimates was conducted by the omission of one study at a time.

The Meta package of R software was applied for the analyses [14].

## Results

### Demographic characteristics of included studies

**Fig. 1:**
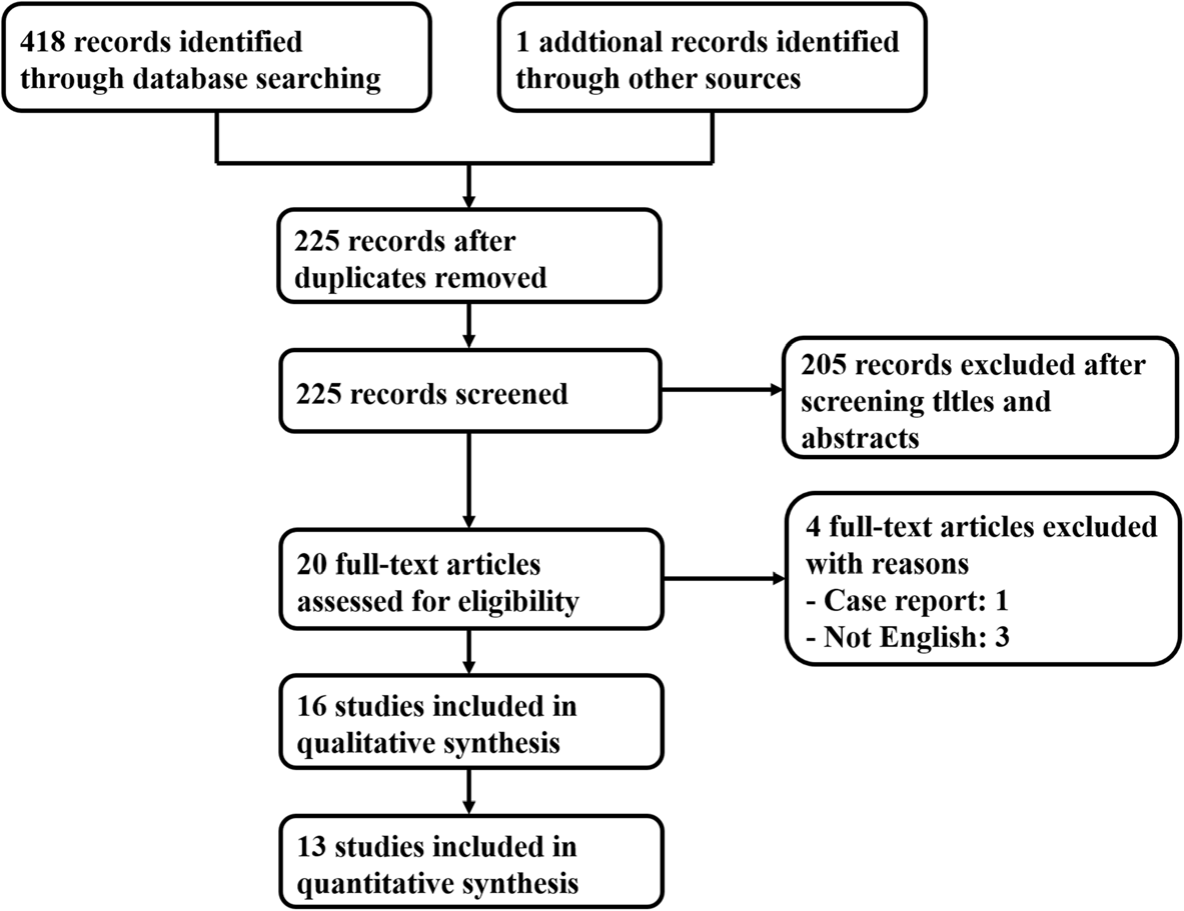
Flow diagram of study selection

The literature selection process is shown in Fig. 1. By searching the databases, 418 studies were found and 1 additional record was identified by reviewing the reference list of related studies. By removing duplicate articles, 225 remained. After screening titles and abstracts, 205 records were excluded. Four full-text were excluded as one was case report and three articles were not English. After full-text screening, 16 studies were considered for qualitative assessment and 13 studies were included for quantitative synthesis [15–30].

All patients were older than 18 years. In 13 studies, 5 RCTs compared the efficacy of PDT with topical corticosteroids. One article clearly stated that patients with the reticular type were included and two articles included the erosive type. The remaining information is presented in Table 1 and Additional file 1: Table S2-S3.

**Table 1:** Characteristics of the studies with lesion response and lesion size changes after PDT

Author	Year	Light sources	Photosensitizer	Lesion types	Administration Method	Lesion locations	Sample size	CR	PR	lesion size of pre-PDT		lesion size of post-PDT	
										Mean	SD	Mean	SD
Aghahosseini F	2006	diode laser	MB	mixed	gargle	mixed	26	4	12	1.8	0.7	1	0.9
Sadaksharam J	2012	xenon lamp	MB	mixed	gargle	mixed	20	0	10				
Sobaniec S	2013	semiconductor laser	chlorin e6 derivative	mixed	topical	mixed	48	14	25	6	4.5	2.7	2.62
						BM/L	40	13	22	6.6	4.63	2.8	2.81
						T/G	8	1	3	3	1.9	2.1	1.21
Prasanna SW	2015	metal halide lamp	MB	mixed	gargle	mixed	15	0	13	3	1.6	0.8	1
Maloth KN	2016	LED	5-ALA	mixed	topical	mixed	10	0	8	2.22	0.79	1.41	0.74
Bakhtiari S	2017	LED	MB	mixed	gargle	mixed	15	0	2				
Mostafa D	2017	diode laser	MB	mixed	gargle	mixed	19	7	9				
Sulewska M	2017	LED	5-ALA	erosive	topical	mixed	22	5	11	1.49	1.45	1.37	1.78
						BM	16	4	7	1.06	0.98	1.08	1.57
						T /G	6	1	4	2.63	1.93	2.13	2.24
Rakesh N	2018	diode laser	5-ALA	erosive	topical	mixed	10	0	10				
Sulewska M	2019	LED	5-ALA	reticular	topical	mixed	124	46	63	3.99	3.73	1.48	1.98
						BM	80	27	44	4.58	4.01	1.67	2.04
						T /G	44	19	19	2.93	2.91	1.13	1.84

*PDT* Photodynamic therapy, *GaAlAs* Gallium-Aluminum-Arsenide, *LED* Light emitting diode, *TB* Toluidine blue, *5-ALA* 5 aminolevulinic acid, *MB* Methylene Blue, *BM/L* Buccal mucosa and/or lips, *T/G* Tongue and/or gingival mucosa; mixed: with different required information or information were not mentioned, *CR* Complete response, *PR* Partial response, *NR* No response, *SD* Standard deviation

### Quality assessment of included studies

The results of the Cochrane Collaboration’s risk of bias assessment and Downs-Black Checklist are shown in Additional file 1: Figure S1 and Additional file 1: Table S4. The included RCT studies had a low or unclear risk of bias and, owing to the wide difference in treatment method between PDT and topical corticosteroids, most did not specify blinding (Additional file 1: Figure S1). The majority of non-RCT studies were of high quality in five fields: study quality, external validity, study bias, confounding effects, and power of the study (Additional file 1: Table S4).

### Data synthesis

#### Lesion response

Ten publications involving 309 lesions assessed the lesion response (CR and PR) after PDT, the details of which are shown in Table 1.

As the recognition criteria for CR was strict, half of the studies had no CR and the pooled proportion of CR by the random effects model (Additional file 1: Figure S2 shows the heterogeneity) was 0.08, which indicated that only 8% of the lesions reached a CR. No publication bias existed (Additional file 1: Figure S3) and the sensitivity analysis showed that the results were relatively robust (Additional file 1: Figure S4).

**Fig. 2:**
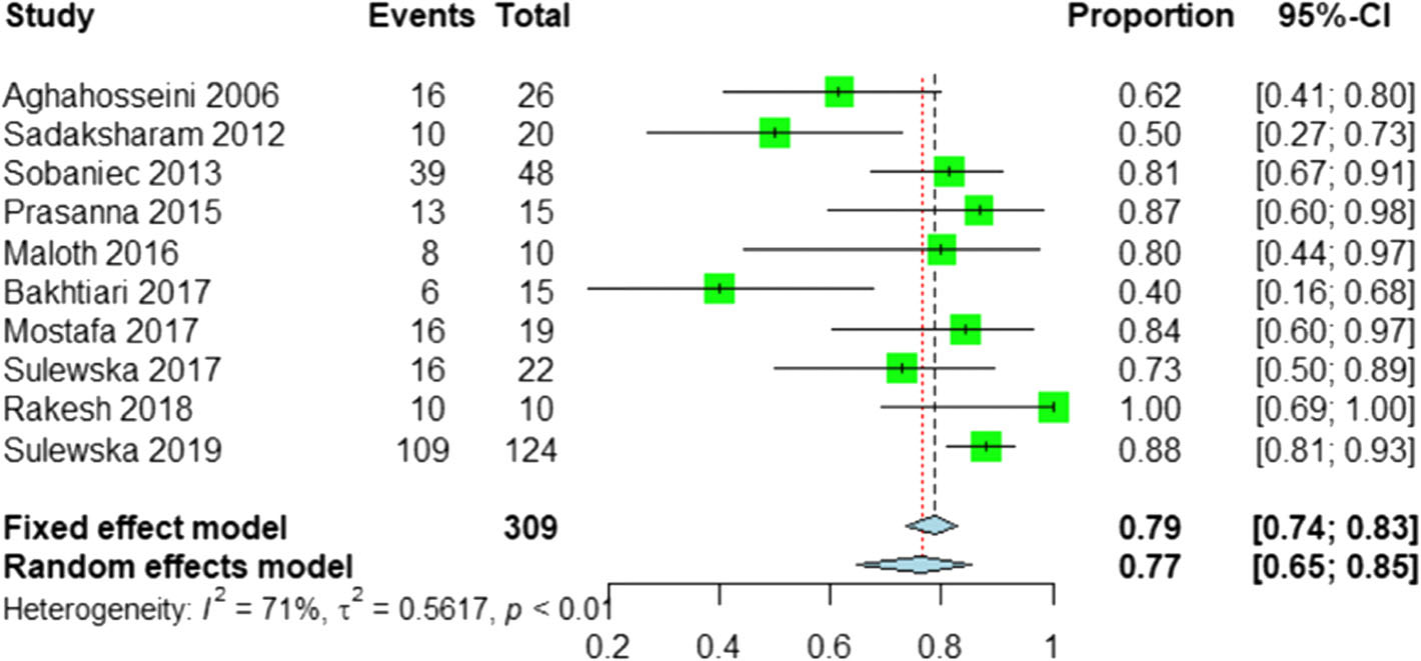
Forest plot of proportions of PR after PDT

The PRs are shown in Fig. 2. As heterogeneity was detected by the *Q* test (*p* value < 0.05) and *I*^*2*^ statistic (71%), the random effects model was applied to pool the overall proportion of PR, which was 0.77 (95% CI: 0.65–0.85). The funnel plot indicated that no publication bias existed (Additional file 1: Figure S5) and the robustness of the results was also validated by sensitivity analysis (Additional file 1: Figure S6).

To examine the influence of factors on the final therapeutic response of PDT, subgroup analyses were performed.

##### Light sources

**Fig. 3:**
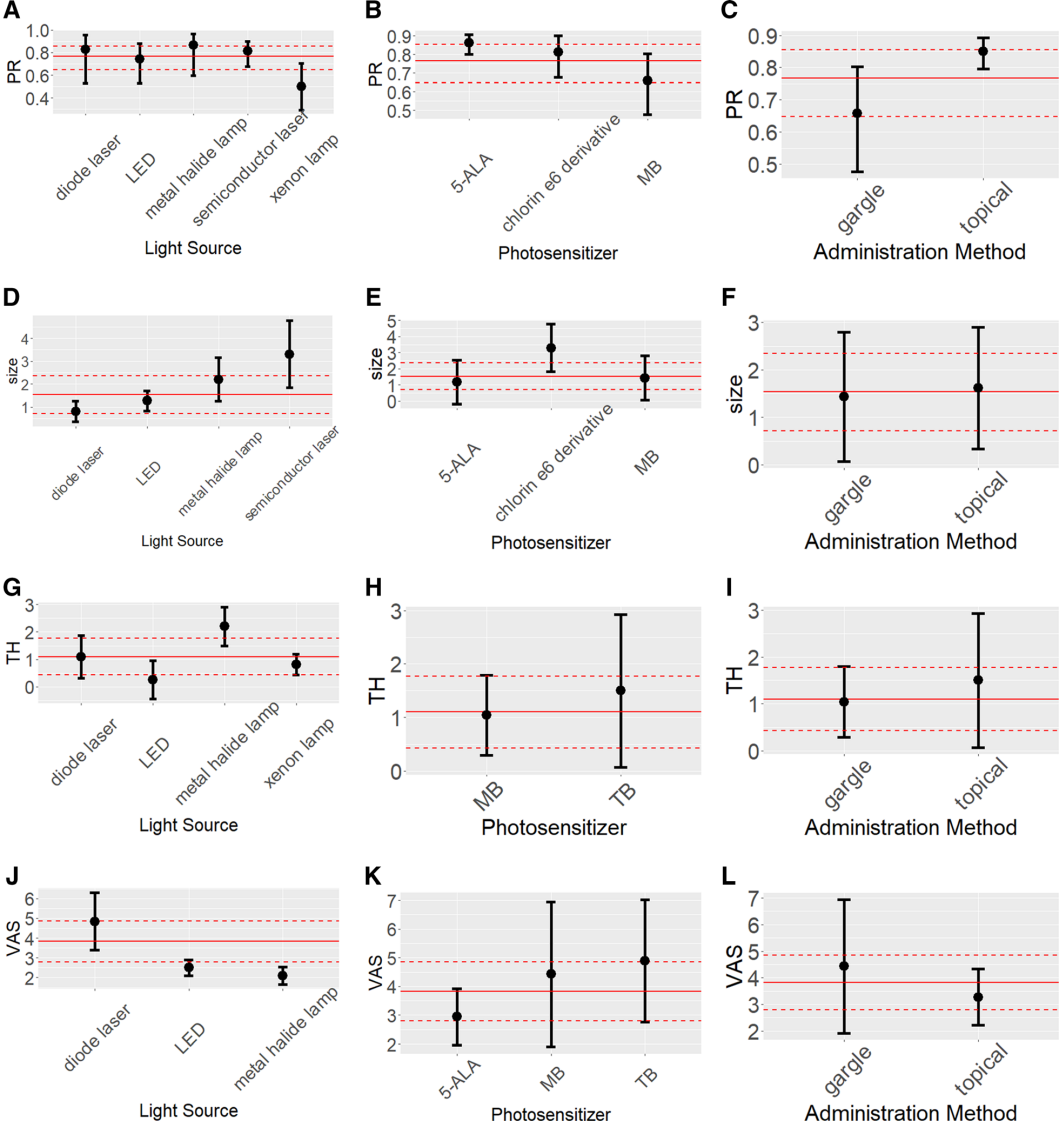
**a-j** showed the results of subgroup analysis with random effects model, three factors were considered for subgroup analysis, namely, light sources (**a**, **d**, **g**, **j**), photosensitizers (**b**, **e**, **h**, **k**), administration methods (**c**, **f**, **i**, **l**). The three plots at the first column represent the results of PR, the plots at the second column represent the results of size, the plots at the third column represent the results of TH, the plots at the fourth column represent the results of VAS. The plots at the third column represent the results of VAS. The full red lines in the plots indicate the pooled overall estimates and the dashed red lines indicate the lower limits and upper limits of their 95% CI

Five types of light source were utilised in 10 studies, including diode laser (three trials), xenon lamp (one trial), semiconductor laser (one trial), metal halide lamp (one trial), and light-emitting diode (LED) (four trials). As the standard of CR was so strict that half of the included studies achieved no CR, we only applied subgroup analysis for the PR. The forest plots of the different light sources are shown in Additional file 1: Figure S7 and Fig. 3a shows the results of the random effects model for the pooled PR. No significant difference (tested by *u* test, *p* > 0.05) was detected in the PR among light sources.

##### Ps

Three types of PS were discussed in the studies, 5-ALA (four trials), MB (five trials), and chlorin e6 derivative (one trial). The pooled PR of 5-ALA was 0.86 (95% CI: 0.80–0.91), which was more effective than other two and the difference was significant (*p* < 0.05) compared to MB (Fig. 3b and Additional file 1: Figure S8).

##### Administration methods

Five trials used topical application and five used gargle administration. Topical application was more effective than the gargle method (*p* < 0.05) and the pooled PR of topical application reached 0.85 (95% CI: 0.80–0.89) (Fig. 3c and Additional file 1: Figure S9).

Additionally, two types of lesion locations (buccal mucosa and/or lips (BM/L) and tongue and/or gingival mucosa (T/G), were detailed in three studies, including 194 lesions. The OR was calculated and pooled to compare the PR of the two lesion locations and the PDT would be regarded as more efficient in BM/L if the OR was greater than 1. The PDT seemed to be more suitable for BM/L, although this was not statistically significant (pooled OR: 1.75, 95% CI: 0.43–7.05) (Additional file 1: Figure S10).

#### Changes of lesions

The variables of lesion size and TH were included to assess the changes of the lesions after PDT.

**Fig. 4:**
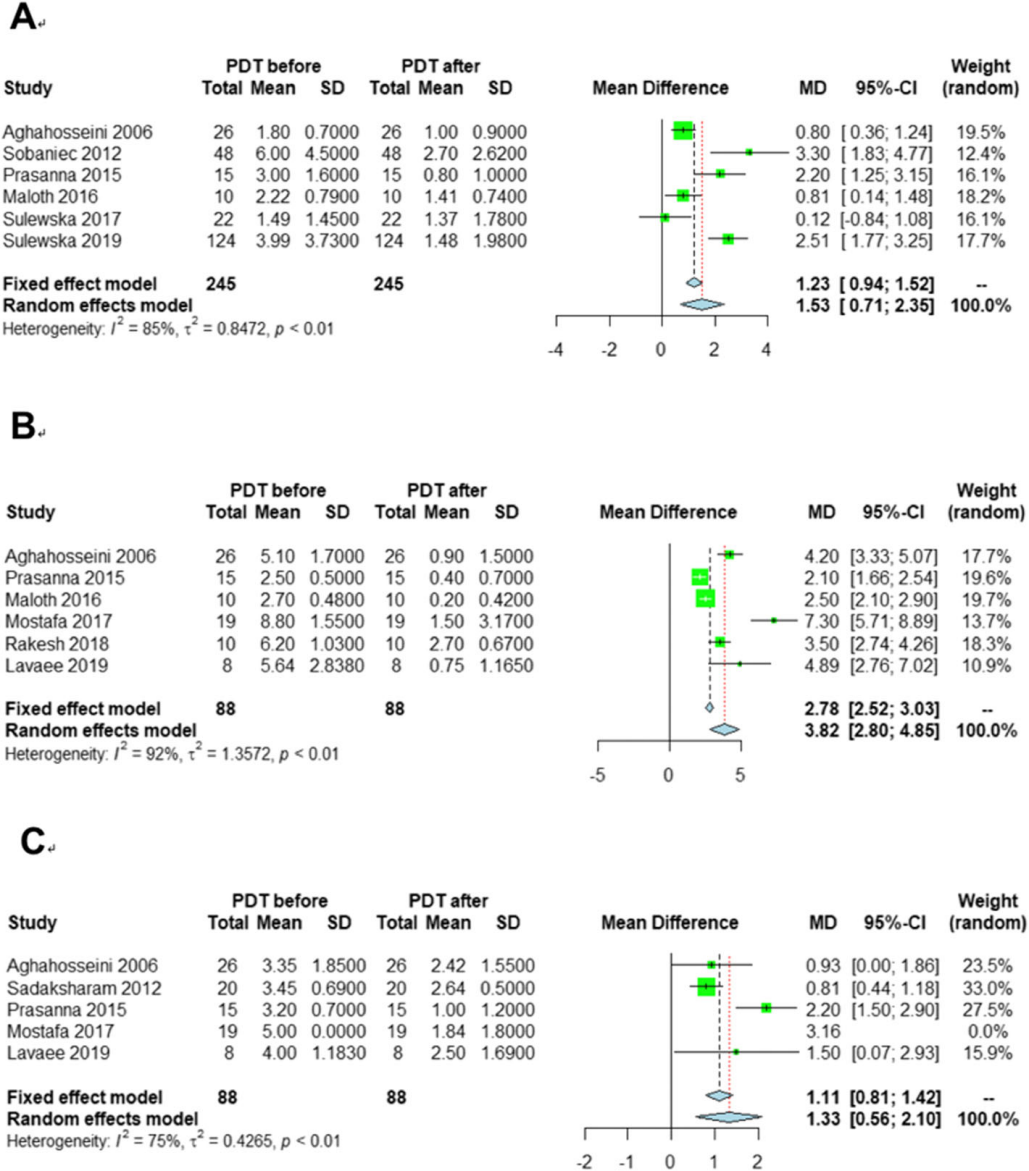
Forest plots of mean difference between before and after PDT in three effect indicators. **a**: lesion size, **b**: TH score, **c**: VAS

The lesion size was recorded in six publications before and after PDT and 245 lesions were identified for meta-analysis. The lesion size decreased by 1.53 cm^2^ (95%: 0.71–2.35) after PDT (Fig. 4a). Heterogeneity existed among the six studies, as the I^2^ statistic was 85% and the *p* value of the *Q* test was lower than 0.01. The sensitivity analysis (Additional file 1: Figure S11) validated that the pooled estimates were stable. Publication bias could be ignored according to the funnel plot (Additional file 1: Figure S12).

Through subgroup analysis, lesion size decreased more using a semiconductor laser than using a diode laser (tested by *u* test, *p* < 0.05, Fig. 3d) and the lesions located on the BM/L decreased more than those located on the T/F/G (pooled MD: 0.37, 95% CI: 0.05–0.68, Additional file 1: Figure S13), whereas no statistically significant differences were detected between PS and administration method (Fig. 3e and Fig. 3f).

To determine the TH score, five trials, including 88 lesions, were involved. Owing to the heterogeneity (Fig. 4b), the random effects model was recommended and the TH score decreased by 1.33 (95% CI: 0.56–2.10) after PDT, which was validated for robustness with the sensitivity analysis (Additional file 1: Figure S14) and publication bias could be ignored (Additional file 1: Figure S15).

The subgroup analysis indicated that the metal halide lamp performed better than the LED and xenon lamp (Fig. 3g), whereas the PS and administration method showed no significant differences in performance (Fig. 3h and Fig. 3i).

#### Clinical pain symptom

The clinical pain symptom was measured by VAS and six studies with 88 lesions used VAS to assess the improvement of pain after PDT. The VAS score decreased by 3.82 (95% CI: 2.80–4.85) **(**Fig. 4c). Heterogeneity existed (*p* value < 0.05 and *I*^*2*^ = 92%) and the result was robust (Additional file 1: Figure S16). Publication bias could be ignored according to the funnel plot (Additional file 1: Figure S17).

Unlike with lesion changes, the subgroup analysis revealed that the efficacy of the diode laser was better than that of the metal halide lamp and LED in relieving pain (*u* test *p* < 0.05, Fig. 3j). No differences were observed in the subgroup analysis of PS and administration method (Fig. 3k and Fig. 3l). Owing to the information shortage, the subgroup analysis of sites of OLP lesions was not performed.

#### Comparison with topical corticosteroids

To obtain an understanding of the efficacy of PDT, five RCT trials with 139 lesions were included to compare PDT with topical corticosteroids. The indicators included PR (recorded in two trials, 68 lesions in total), TH (recorded in four trials, 109 lesions in total), and VAS (recorded in four trials, 109 lesions in total), the details of which are shown in Additional file 1: Table S3. The pooled estimates indicated varied results. The efficacy of PDT was better than that of topical corticosteroids (pooled OR: 6.15, 95% CI: 1.65–22.97) based on the on PR (Additional file 1: Figure S18). With the TH score (Additional file 1: Figure S19), the pooled mean difference was 0.62 (95% CI: − 0.46–1.71), which indicated that the two treatments had similar efficacy to decrease lesion size. Additionally, the pooled mean difference of VAS (Additional file 1: Figure S20) was − 0.30 (95% CI: − 1.99–1.40), which indicated the similar improvement of pain between the two treatments.

### Other factors in PDT

The wavelength of 630–660 nm and energy density of 80–150 J/cm^2^ were commonly used. The duration of irradiation ranged between 120 s and 600 s. The range of dressing time was 5–120 min. The frequency of PDT application ranged from 1 to 10 times throughout the study period at 1- to 2-week intervals. The details are shown in Table 2.

**Table 2:** Parameters without meta-analysis of the studies included

Author	Year	Wavelength (nm)	Energy density (J/cm^2^)	Duration of irradiation(s)	Dressing time (min)	Frequency of PDT	Recurrence	Follow-up time (month)
Aghahosseini, F	2006	632	120	120	5	1 session	NA	3
Sadaksharam J	2012	632 ± 5	120	1200	5	4 weekly	NA	6
Sobaniec S	2012	660	90	NA	60	2 weekly	NA	5
Kvaal SI	2013	600–660	75	NA	60	1 session	2	6–48
Saleh WE	2014	660	100–130	120	5	1 session	NA	1
Jajarm HH	2015	630	1.5	150	10	Once 2-weeks	0	1
Prasanna SW	2015	630 ± 10	120	NA	5	Once a week	NA	3
Maloth KN	2016	420	210	600	30	1 session	NA	1
Bakhtiari S	2017	630	7.2–14.4	120	10	NA	NA	3
Mostafa D	2017	660	100–130	70	NA	Once a week	0	2
Sulewska M	2017	630	150	500	120	10 weekly	4	12
Mirza S	2018	630	1.5	150	10	Twice a week	NA	1
Paiziyeva Z	2018	632.7	NA	NA	NA	NA	NA	NA
Rakesh N	2018	600–670	80	NA	120	1 session	0	48
Lavaee F	2019	660	19.23	600	10	3 sessions	NA	2
Sulewska M	2019	630	150	500	120	10 weekly	0	12

*nm* Nanometers, *J/cm*^*2*^ Joules per square centimeters, *NA* Not available

The majority of patients experienced no discomfort or only minor adverse effects (pain, mild burning sensation) during treatment, which disappeared immediately. Most studies were conducted with a usual follow-up time of 1–12 months. Among all studies, six patients in two studies were reported to have relapsed after PDT. However, most studies did not report a cancerous patient.

## Discussion

The pooled estimates of lesion response, changes in size, VAS, and TH revealed that PDT could not only reduce the lesion size but also reduce pain. PDT is a new non-invasive treatment that could be effective for the treatment of OLP.

The topical use of 5-ALA had a higher efficacy than gargling MB in terms of PR. The relatively poor outcome of MB could be owing to the short gargling time of 5 min. The time of the topical use of 5-ALA can be 30-–120 min. The longer the PS stays on the lesions, the better efficacy of PDT. Constant saliva secretion and frequent tissue movement may impair drug absorption. Thus, a high local concentration of PS may achieve better potency than the use of drugs. In four studies that used 5-ALA as the PS, the range of 5-ALA was 4–5%. Therefore, the topical use of 5% ALA may be recommended as the optimal modality.

When 5% ALA is used a wavelength of 630 nm is recommended because 635 nm corresponds to the absorption peak of 5-ALA. In studies that involve gargling MB, the chosen wavelength of 632–660 nm did not reach the maximum absorption wavelength of MB (around 665 nm), which also partially explains why the effect of MB was less than that of 5-ALA. Therefore, it is important to choose a suitable wavelength for PS.

In terms of VAS, the diode laser showed a better clinical PR in the treatment of OLP, perhaps because it emits only one wavelength of light. Thus, we recommend the diode laser as the first option to relieve pain. However, to change lesion size, the efficacy of the semiconductor laser was higher than that of the diode laser.

Some scholars [26] have supported the hypothesis that PDT stimulates healing processes, which become more evident during long-term observation, particularly within the masticatory mucosa. This tentative hypothesis needs to be confirmed by a greater number of cured cases. In the study of Sulewska et al. [23], the mean lesion size reduction was 62.91%, which was significant, after PDT, showing a slightly higher value for the lesions on the BM/L (63.54%) than on the T/G (61.43%), whereas in our study, the effect of PDT on the lesions on the BM/L and T/F/G was similar.

A previous study has compared the apoptosis level in reticular and erosive OLP showed significantly more apoptosis and a markedly lower thickness of the oral epithelium in the erosive type than in the reticular type, which indicated more inflammation and cell destruction in erosive OLP than in reticular OLP [31]. PS tends to accumulate in abnormal hyperplasia and tumour tissue and some researchers believe that this may be related to a defect in the cell membrane structure. We speculated that the PDT of erosive OLP is more effective than that of reticular OLP. However, the subgroup analysis of disease type in our study showed no statistical significance based on the *u* test, possibly because two studies were included for erosive OLP and only one for reticular OLP.

The PDT of OLP resulted in fewer adverse reactions. The majority of patients experienced no discomfort or only minor adverse effects (pain, mild burning sensation) during treatment, which disappeared immediately.

Currently, the recurrence rate of OLP after PDT is unknown but one feature of OLP is easy recurrence. Among all studies, six patients in two studies experience OLP recurrence after PDT but three studies reported no recurrence in 1–12 months follow-up. OLP is a chronic disease; thus, the follow-up periods need to be longer to reliably determine recurrence rates after PDT. PDT can reduce the risk of malignant transformation. One study has revealed that the malignant transformation rate of OLP is 1.4%; however, the studies in this review did not record this rate [3]. Thus, the long-term effects of PDT remain unclear and there is an urgent need to carry out large sample, multi-centre, clinical research to explore and verify the factors that influence the efficacy of PDT.

Presently, the most common treatment for OLP is topical corticosteroids [4]. We compared the efficacy of PDT to topical corticosteroids. A similar efficacy was observed between PDT and corticosteroid therapy. PDT had fewer side effects than steroids. Therefore, PDT can be used as an optional treatment method for resistant or recurrent OLP.

A few weaknesses of this study need to be addressed in the future. An insufficient number of trials met the inclusion criteria, which reduced the significance of the results, especially in the subgroup analysis and comparison with topical corticosteroids. The outcome measures varied in the different trials, which hindered data combination. Additionally, heterogeneity in some parameters, such as wavelength and energy density, may have led to low statistical power. Although these disadvantages existed in this study, the results still provide clinicians with a comprehensive view of the efficacy of PDT in OLP, although more high-quality clinical studies are required to improve the reliability of the results.

## Conclusions

PDT could be a valuable optional treatment for the management of OLP. The overall PR could reach 0.75 and the topical use of 5% ALA could be the optimal PS. These results indicated that the curative effect of PDT is similar to that of topical corticosteroids in the treatment of OLP.

## Supplementary information

**Additional file 1: **Appendix **Table S1.** PRISMA checklist. Appendix **Table S2.** Characteristics of the 16 studies included for qualitative assessment. Appendix **Table S3.** Results of bias risk assessment for each included non-RCT (score). Appendix **Table S4.** Characteristics of the studies with VAS and TH changes after treatment. Appendix **Figure S1.** Risk of bias summary: review authors’ judgments about each risk of bias items for each included RCT. Appendix **Figure S2.** Forest plot of CR after PDT. Appendix **Figure S3.** Funnel plot of CR after PDT. Appendix **Figure S4.** Sensitivity analysis for CR after PDT. Appendix **Figure S5.** Funnel plot of PR after PDT. Appendix **Figure S6.** Sensitivity analysis for PR after PDT. Appendix **Figure S7.** Forest plots of PR after PDT: subgroup analysis of light sources. Appendix **Figure S8.** Forest plots of PR after PDT: subgroup analysis of photosensitizers. Appendix **Figure S9.** Forest plots of PR after PDT: subgroup analysis of administration methods. Appendix **Figure S10.** Forest plot of PR after PDT: subgroup analysis of lesion locations. Appendix **Figure S11.** Sensitivity analysis for the changes of lesion size. Appendix **Figure S12.** Funnel plot for lesion size after PDT. Appendix **Figure S13.** Forest plot of size after PDT: subgroup analysis of lesion location. Appendix **Figure S14.** Sensitivity analysis for the results of TH. Appendix **Figure S15.** Funnel plot for TH after PDT. Appendix **Figure S16.** Sensitivity analysis for the results of VAS. Appendix **Figure S17.** Funnel plot for VAS after PDT. Appendix **Figure S18.** Forest plot of PDT comparing with topical corticosteroids on PR. Appendix **Figure S19.** Forest plot of PDT comparing with topical corticosteroids on TH. Appendix **Figure S20.** Forest plot of PDT comparing with topical corticosteroids on VAS.

## Data Availability

All data generated or analysed during this study are included in this published article.

## Abbreviations

5-ALA: 5-aminolevulinic acid
BM/L: Buccal mucosa and/or lips
CI: Confidence interval
CR: Complete response
LED: Light-emitting diode
MB: Methylene blue
OLP: Oral lichen planus
OPMD: Oral potentially malignant disorder
OR: Odds ratio
PDT: Photodynamic therapy
PR: Partial response
PROSPERO: Prospective Register of Systematic Reviews
PS: Photosensitiser
RCT: Randomised controlled trial
T/G: Tongue and/or gingival mucosa
TH: Thongprasom sign
VAS: Visual analogue scale
