# Supplementary material for: Efficacy evaluation of photodynamic therapy for oral lichen planus: a systematic review and meta-analysis
Source: BMC Oral Health. 2020 Nov 4;20:302. doi: 10.1186/s12903-020-01260-x (PMC7640434; doi:10.1186/s12903-020-01260-x)
Supplement: Supplementary file 1 — Additional file 1: Appendix Table S1. PRISMA checklist. Appendix Table S2. Characteristics of the 16 studies included for qualitative assessment. Appendix Table S3. Results of bias risk assessment for each included non-RCT (score). Appendix Table S4. Characteristics of the studies with VAS and TH changes after treatment. Appendix Figure S1. Risk of bias summary: review authors’ judgments about each risk of bias items for each included RCT. Appendix Figure S2. Forest plot of CR after PDT. Appendix Figure S3. Funnel plot of CR after PDT. Appendix Figure S4. Sensitivity analysis for CR after PDT. Appendix Figure S5. Funnel plot of PR after PDT. Appendix Figure S6. Sensitivity analysis for PR after PDT. Appendix Figure S7. Forest plots of PR after PDT: subgroup analysis of light sources. Appendix Figure S8. Forest plots of PR after PDT: subgroup analysis of photosensitizers. Appendix Figure S9. Forest plots of PR after PDT: subgroup analysis of administration methods. Appendix Figure S10. Forest plot of PR after PDT: subgroup analysis of lesion locations. Appendix Figure S11. Sensitivity analysis for the changes of lesion size. Appendix Figure S12. Funnel plot for lesion size after PDT. Appendix Figure S13. Forest plot of size after PDT: subgroup analysis of lesion location. Appendix Figure S14. Sensitivity analysis for the results of TH. Appendix Figure S15. Funnel plot for TH after PDT. Appendix Figure S16. Sensitivity analysis for the results of VAS. Appendix Figure S17. Funnel plot for VAS after PDT. Appendix Figure S18. Forest plot of PDT comparing with topical corticosteroids on PR. Appendix Figure S19. Forest plot of PDT comparing with topical corticosteroids on TH. Appendix Figure S20. Forest plot of PDT comparing with topical corticosteroids on VAS. [file 12903_2020_1260_MOESM1_ESM.docx]

**Supplemental Appendices**

Yuqing He, Jiaxin Deng, Yi Zhao, et al. Efficacy evaluation of photodynamic therapy for oral lichen planus: a systematic review and meta-analysis

**Appendix Tables:**

Appendix Table 1. PRISMA checklist.

Appendix Table 2. Characteristics of the 16 studies included for qualitative assessment

Appendix Table 3. Results of bias risk assessment for each included non-RCT (score).

Appendix Table 4. Characteristics of the studies with VAS and TH changes after treatment.

**Appendix Figures:**

Appendix Fig. 1. Risk of bias summary: review authors’ judgments about each risk of bias items for each included RCT.

Appendix Fig. 2. Forest plot of CR after PDT.

Appendix Fig. 3. Funnel plot of CR after PDT.

Appendix Fig. 4. Sensitivity analysis for CR after PDT.

Appendix Fig. 5. Funnel plot of PR after PDT.

Appendix Fig. 6. Sensitivity analysis for PR after PDT.

Appendix Fig. 7. Forest plots of PR after PDT: subgroup analysis of light sources.

Appendix Fig. 8. Forest plots of PR after PDT: subgroup analysis of photosensitizers.

Appendix Fig. 9. Forest plots of PR after PDT: subgroup analysis of administration methods.

Appendix Fig. 10. Forest plot of PR after PDT: subgroup analysis of lesion locations

Appendix Fig. 11. Sensitivity analysis for the changes of lesion size.

Appendix Fig. 12. Funnel plot for lesion size after PDT.

Appendix Fig. 13. Forest plot of size after PDT: subgroup analysis of lesion location.

Appendix Fig. 14. Sensitivity analysis for the results of TH.

Appendix Fig. 15. Funnel plot for TH after PDT.

Appendix Fig. 16. Sensitivity analysis for the results of VAS.

Appendix Fig. 17. Funnel plot for VAS after PDT.

Appendix Fig. 18. Forest plot of PDT comparing with topical corticosteroids on PR

Appendix Fig. 19. Forest plot of PDT comparing with topical corticosteroids on TH

Appendix Fig. 20. Forest plot of PDT comparing with topical corticosteroids on VAS

**This supplementary material has been provided by the authors to give readers additional information about this network meta-analysis.**

**Appendix Table 1. PRISMA checklist.**

| **Section/topic** | **#** | **Checklist item** | **Reported on page #** |
| --- | --- | --- | --- |
| **TITLE** | | |  |
| Title | 1 | Identify the report as a systematic review, meta-analysis, or both. | 1 |
| **ABSTRACT** | | |  |
| Structured summary | 2 | Provide a structured summary including, as applicable: background; objectives; data sources; study eligibility criteria, participants, and interventions; study appraisal and synthesis methods; results; limitations; conclusions and implications of key findings; systematic review registration number. | 1-2 |
| **INTRODUCTION** | | |  |
| Rationale | 3 | Describe the rationale for the review in the context of what is already known. | 3 |
| Objectives | 4 | Provide an explicit statement of questions being addressed with reference to participants, interventions, comparisons, outcomes, and study design (PICOS). | 3-4 |
| **METHODS** | | |  |
| Protocol and registration | 5 | Indicate if a review protocol exists, if and where it can be accessed (e.g., Web address), and, if available, provide registration information including registration number. | 4 |
| Eligibility criteria | 6 | Specify study characteristics (e.g., PICOS, length of follow-up) and report characteristics (e.g., years considered, language, publication status) used as criteria for eligibility, giving rationale. | 4 |
| Information sources | 7 | Describe all information sources (e.g., databases with dates of coverage, contact with study authors to identify additional studies) in the search and date last searched. | 4 |
| Search | 8 | Present full electronic search strategy for at least one database, including any limits used, such that it could be repeated. | 4 |
| Study selection | 9 | State the process for selecting studies (i.e., screening, eligibility, included in systematic review, and, if applicable, included in the meta-analysis). | 4 |
| Data collection process | 10 | Describe method of data extraction from reports (e.g., piloted forms, independently, in duplicate) and any processes for obtaining and confirming data from investigators. | 4-5 |
| Data items | 11 | List and define all variables for which data were sought (e.g., PICOS, funding sources) and any assumptions and simplifications made. | 4-5 |
| Risk of bias in individual studies | 12 | Describe methods used for assessing risk of bias of individual studies (including specification of whether this was done at the study or outcome level), and how this information is to be used in any data synthesis. | 5 |
| Summary measures | 13 | State the principal summary measures (e.g., risk ratio, difference in means). | 5-6 |
| Synthesis of results | 14 | Describe the methods of handling data and combining results of studies, if done, including measures of consistency (e.g., I^2^) for each meta-analysis. | 5-6 |
| Risk of bias across studies | 15 | Specify any assessment of risk of bias that may affect the cumulative evidence (e.g., publication bias, selective reporting within studies). | 5 |
| Additional analyses | 16 | Describe methods of additional analyses (e.g., sensitivity or subgroup analyses, meta-regression), if done, indicating which were pre-specified. | 5-6 |
| **RESULTS** | | |  |
| Study selection | 17 | Give numbers of studies screened, assessed for eligibility, and included in the review, with reasons for exclusions at each stage, ideally with a flow diagram. | 6-7 |
| Study characteristics | 18 | For each study, present characteristics for which data were extracted (e.g., study size, PICOS, follow-up period) and provide the citations. | 6 |
| Risk of bias within studies | 19 | Present data on risk of bias of each study and, if available, any outcome level assessment (see item 12). | Additional file 1 |
| Results of individual studies | 20 | For all outcomes considered (benefits or harms), present, for each study: (a) simple summary data for each intervention group (b) effect estimates and confidence intervals, ideally with a forest plot. | 7-14 |
| Synthesis of results | 21 | Present results of each meta-analysis done, including confidence intervals and measures of consistency. | 7-14 |
| Risk of bias across studies | 22 | Present results of any assessment of risk of bias across studies (see Item 15). | Additional file 1 |
| Additional analysis | 23 | Give results of additional analyses, if done (e.g., sensitivity or subgroup analyses, meta-regression [see Item 16]). | 7-14 |
| **DISCUSSION** | | |  |
| Summary of evidence | 24 | Summarize the main findings including the strength of evidence for each main outcome; consider their relevance to key groups (e.g., healthcare providers, users, and policy makers). | 14-16 |
| Limitations | 25 | Discuss limitations at study and outcome level (e.g., risk of bias), and at review-level (e.g., incomplete retrieval of identified research, reporting bias). | 16 |
| Conclusions | 26 | Provide a general interpretation of the results in the context of other evidence, and implications for future research. | 16 |
| **FUNDING** | | |  |
| Funding | 27 | Describe sources of funding for the systematic review and other support (e.g., supply of data); role of funders for the systematic review. | 17 |

**Appendix Table 2. Characteristics of all the 16 studies included.**

| Author Year | Intervention (PDT) | | | | | Control (TC) | | Endpoints included in meta-analysis |
| --- | --- | --- | --- | --- | --- | --- | --- | --- |
|  | Gender (M/F) (n) | Age (years) | Light sources | Photosensitizer | Administration Method | Gender (M/F) (n) | Age (years) |  |
| Aghahosseini F 2006 | 1/12 | mean=42.5 | diode laser | MB | gargle | NA | NA | lesion response; lesion size changes; VAS; TH |
| Sadaksharam J 2012 | 7/13 | 22-58 | xenon lamp | MB | gargle | NA | NA | lesion response |
| Sobaniec S 2013 | 6/17 | 31-82 | semiconductor laser | chlorin e6 derivative | topical | NA | NA | lesion response; lesion size changes |
| Kvaal SI 2013 | NA | NA | red light | MAL | topical | NA | NA | NA |
| Saleh WE 2014 | NA | 30-60 | focal red light | MB | NA | NA | 30-60 | NA |
| Jajarm HH 2015 | 3/8 | 48.71±13.53 | GaAlAs laser | TB | topical | 5/9 | 43.73±10.01 | VAS; TH |
| Prasanna SW 2015 | 4/2 | 20-80 | metal halide lamp | MB | gargle | NA | NA | lesion response; lesion size changes; VAS; TH |
| Maloth KN 2016 | NA | 33.60±9.28 | LED | 5-ALA | topical | NA | NA | lesion response; lesion size changes; VAS |
| Bakhtiari S 2017 | NA | mean=47.2 | LED | MB | gargle | NA | mean=53.4 | lesion response; TH |
| Mostafa D 2017 | 2/8 | 48.6±5.25 | diode laser | MB | gargle | 1/9 | 47.0 ± 6.25 | lesion response; VAS; TH |
| Sulewska M 2017 | 0/12 | 69.63 ± 6.03 | LED | 5-ALA | topical | NA | NA | lesion response; lesion size changes |
| Mirza S 2018 | 3/12 | 52.6±11.4 | GaAlAs laser | TB | topical | 4/11 | 49.2±10.6 | VAS |
| Paiziyeva Z 2018 | 14/26 | 30-66 | LEDs |  | NA | NA | NA | NA |
| Rakesh N 2018 | 2/8 | 47.9±10.65 | diode laser | 5-ALA | topical | NA | NA | lesion response; VAS |
| Lavaee F 2019 | NA | NA | diode laser | TB | topical | NA | NA | VAS; TH |
| Sulewska M 2019 | 14/36 | 26–84 | LED | 5-ALA | topical | NA | NA | lesion response; lesion size changes |

PDT: photodynamic therapy; TC: topical corticosteroids; M: male; F: female; GaAlAs: Gallium-Aluminum-Arsenide; LED: light emitting diode; TB: toluidine blue; 5-ALA: 5 aminolevulinic acid; MB: Methylene Blue；MAL: Methyl 5-aminolevulinate; NA: not available; VAS: visual analogue scale; TH: Thongprasom score

**Appendix Table 3. Characteristics of the studies with VAS and TH changes after treatment.**

| Author Year | Light sources | Photosensitizer | Lesion types | Administration method | Intervention (n) | Control (n) | VAS (Mean ± SD) | | | | TH (Mean ± SD) | |
| --- | --- | --- | --- | --- | --- | --- | --- | --- | --- | --- | --- | --- |
|  |  |  |  |  |  |  | Pre-PDT;  Post-PDT | Pre-TC;  Post-TC | Difference  pre-post PDT | Difference  pre-post TC | Pre-PDT;  Post-PDT | Pre-TC;  Post-TC |
| Lavaee F 2019 | diode laser | TB | mixed | topical short | PDT (n=8) | TC (n=8) | 5.64 ± 2.838;  0.75 ± 1.165 | 5.82 ±3.157;  1.38 ±2.504 | 5.00 ± 3.162 | 4.12 ± 2.1 | 4 ± 1.183;  2.5 ± 1.69 | 3.45 ±1.036;  2.25 ±1.165 |
| Mirza S 2018 | GaAlAs laser | TB | mixed | topical short | PDT (n=15) | TC (n=15) |  |  | 21.4% ± 14.8% | 48.5% ± 17.3% |  |  |
| Bakhtiari S 2017 | LED | MB | mixed | gargle | PDT (n=15) | TC (n=15) |  |  |  |  | 3.53 ± 1.06;  3.27 ± 0.88 |  |
| Jajarm HH 2015 | GaAlAs laser | TB | mixed | topical short | PDT (n=11) | TC (n=14) |  |  | 25.09% ± 15.4% | 53.71% ± 18.63% | 3.45 ± 0.69;  2.64 ± 0.5 | 3.07 ± 0.83;  2.07 ± 0.62 |
| Rakesh N 2018 | diode laser | 5-ALA | erosive | topical | PDT (n=10) |  | 6.2 ± 1.03;  2.7 ± 0.67 |  |  |  |  |  |
| Aghahosseini F 2006 | diode laser | MB | mixed | gargle | PDT (n=26) |  | 5.1 ± 1.7;  0.9 ± 1.5 |  |  |  | 3.35 ± 1.85;  2.42 ± 1.55 |  |
| Prasanna SW 2015 | metal halide lamp | MB | mixed | gargle | PDT (n=15) |  | 2.5±0.5;  0.4 ± 0.7 |  |  |  | 3.2 ± 0.7  1 ± 1.2 |  |
| Mostafa D 2017 | diode laser | MB | mixed | gargle | PDT (n=19) | TC (n=19) | 8.8 ± 1.55;  1.5 ± 3.17 | 8.7 ±1.16;  5.8 ± 3.43 |  |  | 5 ± 0;  1.84 ± 1.8 | 4.79 ± 0.42;  3.79 ± 2.04 |
| Maloth KN 2016 | LED | 5-ALA | mixed | Topical short | PDT (n=10) |  | 2.7 ± 0.48;  0.2 ± 0.42 |  |  |  |  |  |

VAS: visual analogue scale; TH: Thongprasom score; TB: toluidine blue; MB: Methylene Blue; ALA: aminolevulinic acid; Topical short: topical applied for no longer than 30 min; Topical long: topical applied for longer than 30 min; PDT: photodynamic therapy; TC: topical corticosteroids; SD: Standard Deviance

**Appendix Table 4.** **Results of bias risk assessment for each included non-RCT (score).**

| Included studies | Reporting | External validity | Bias | Confounding | Power | Overall score |
| --- | --- | --- | --- | --- | --- | --- |
| Rakesh 2018 | 7 | 0 | 5 | 1 | 5 | 18 |
| Prasanna 2015 | 7 | 2 | 5 | 1 | 5 | 20 |
| Aghahosseini 2006 | 7 | 0 | 5 | 1 | 5 | 18 |
| Sadaksharam 2012 | 7 | 2 | 5 | 1 | 5 | 20 |
| Kvaal 2013 | 7 | 0 | 5 | 1 | 5 | 18 |
| Maloth 2016 | 7 | 1 | 5 | 3 | 5 | 21 |
| Sobaniec 2012 | 7 | 2 | 5 | 1 | 5 | 20 |
| Sulewska 2017 | 7 | 0 | 5 | 1 | 5 | 18 |
| Sulewska 2019 | 7 | 0 | 5 | 1 | 5 | 18 |
| Saleh 2014 | 7 | 2 | 5 | 3 | 5 | 22 |
| Paiziyeva 2018 | 7 | 0 | 4 | 1 | 5 | 17 |


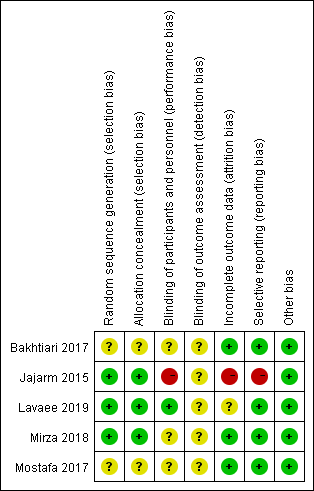


**Appendix Fig. 1** Risk of bias summary: review authors’ judgments about each risk of bias items for each included RCT.


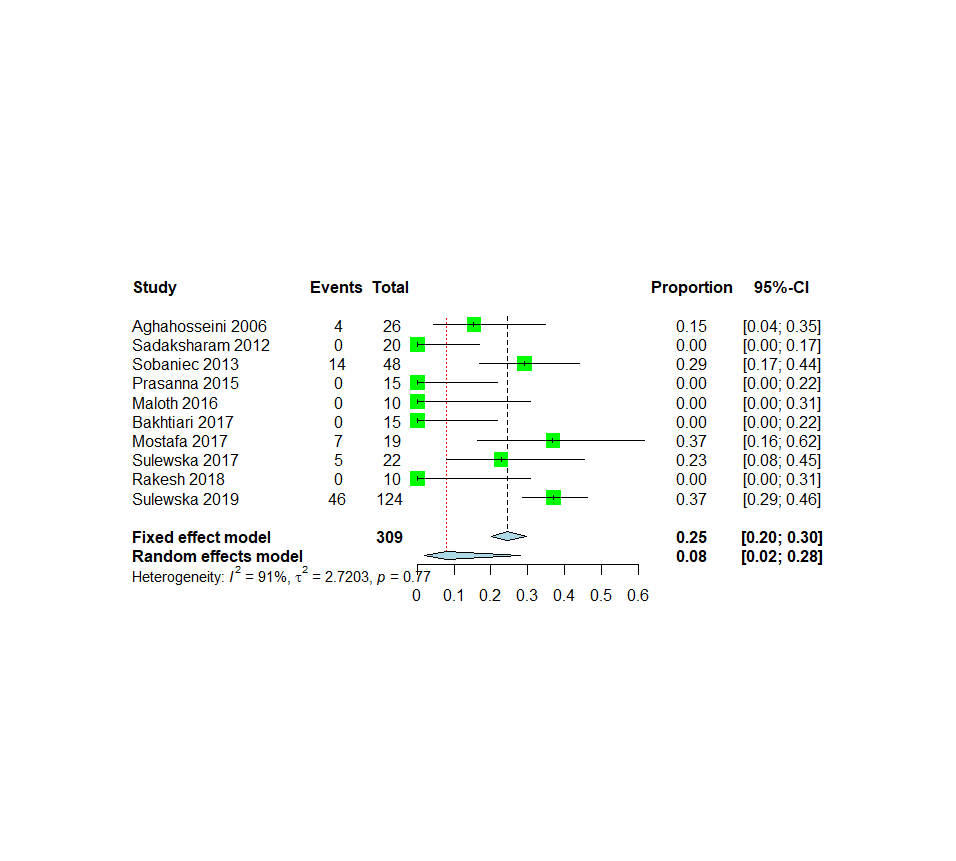


**Appendix Fig. 2** Forest plot of CR after PDT


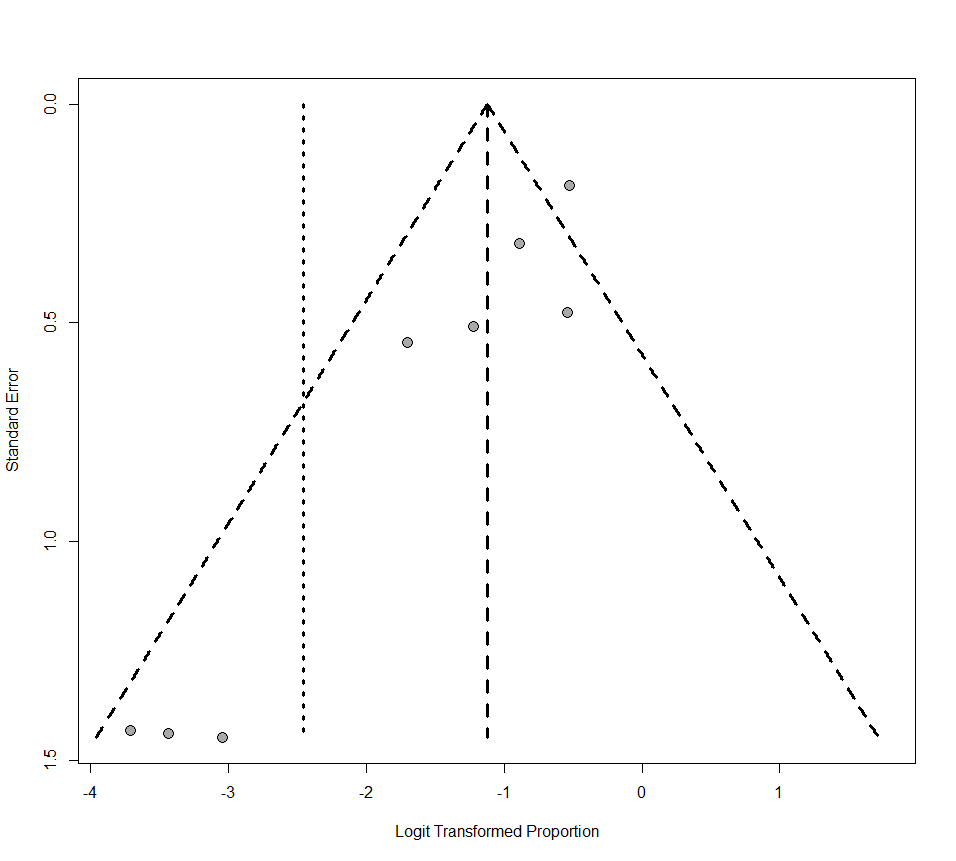


**Appendix Fig. 3** Funnel plot of CR after PDT


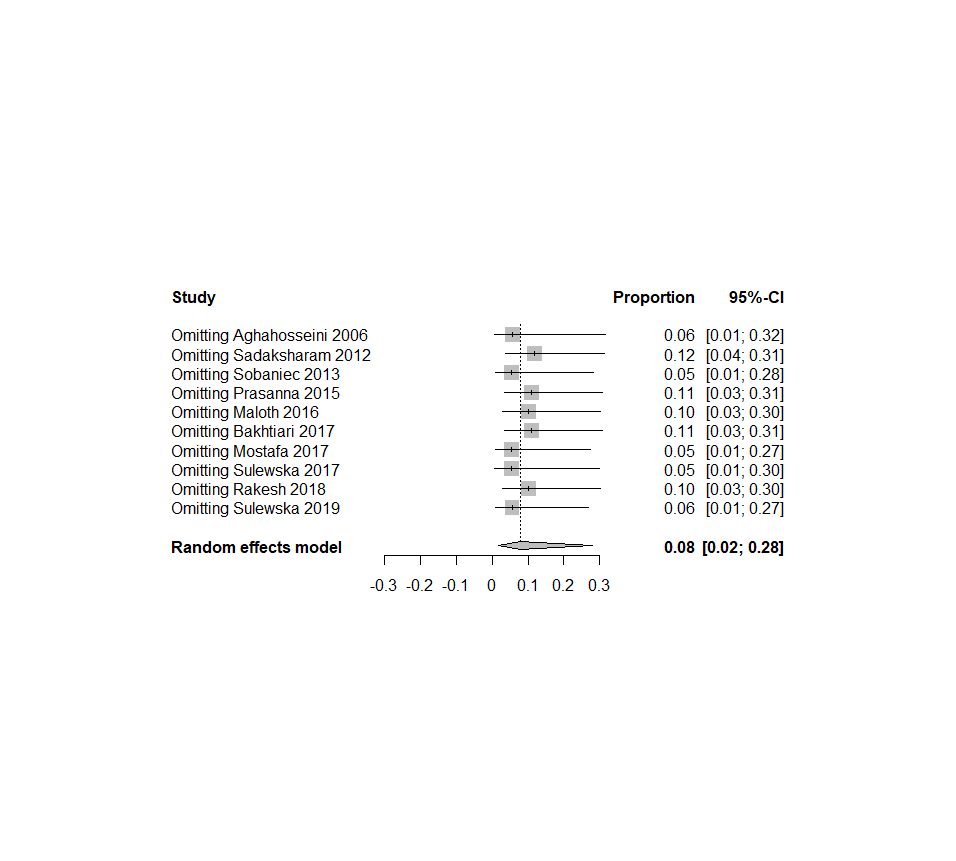


**Appendix Fig. 4**. Sensitivity analysis for CR after PDT


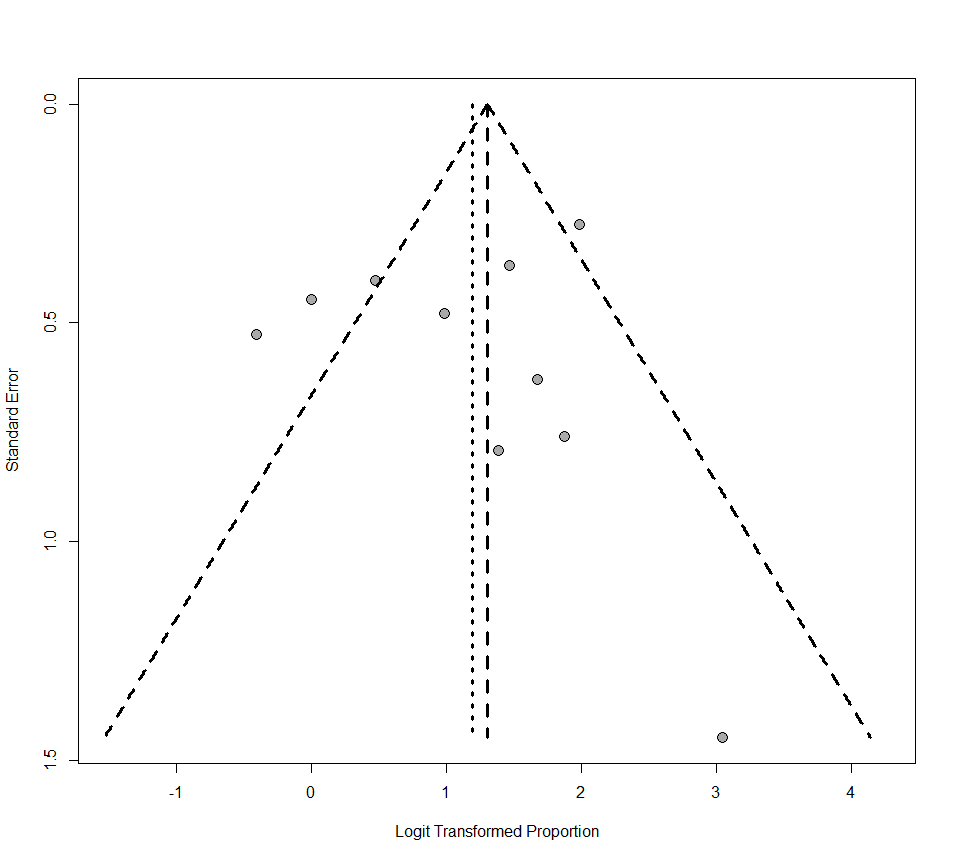


**Appendix Fig.5** Funnel plot of PR after PDT


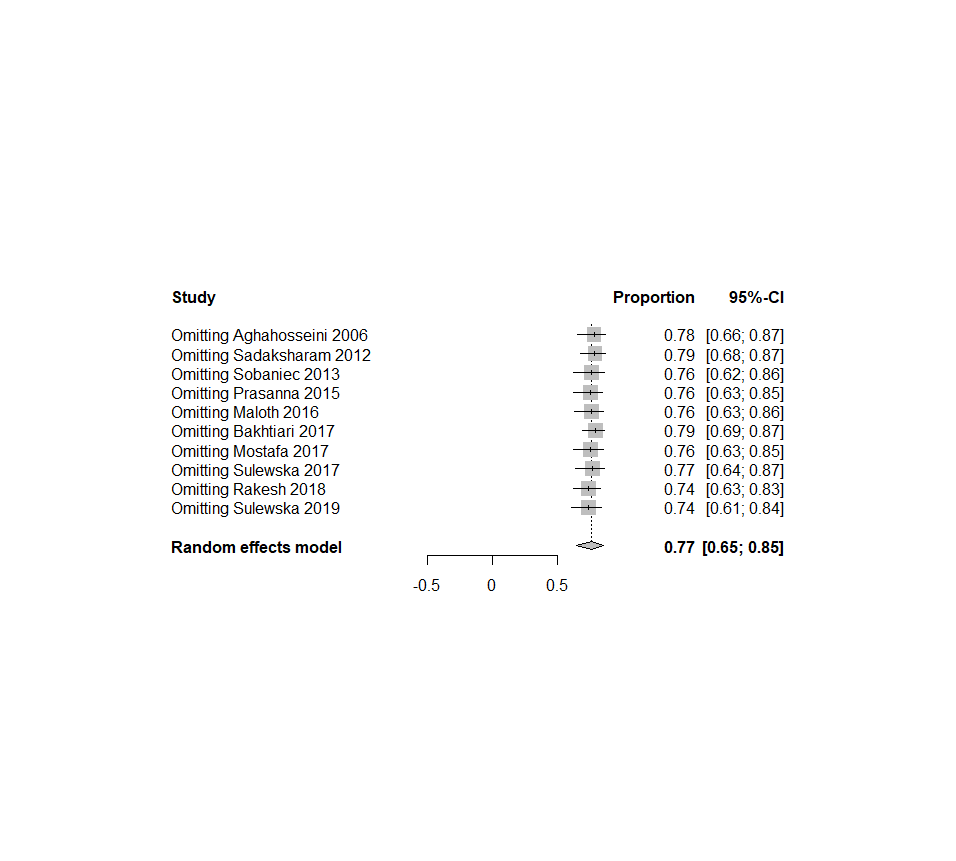


**Appendix Fig. 6**. Sensitivity analysis for PR after PDT


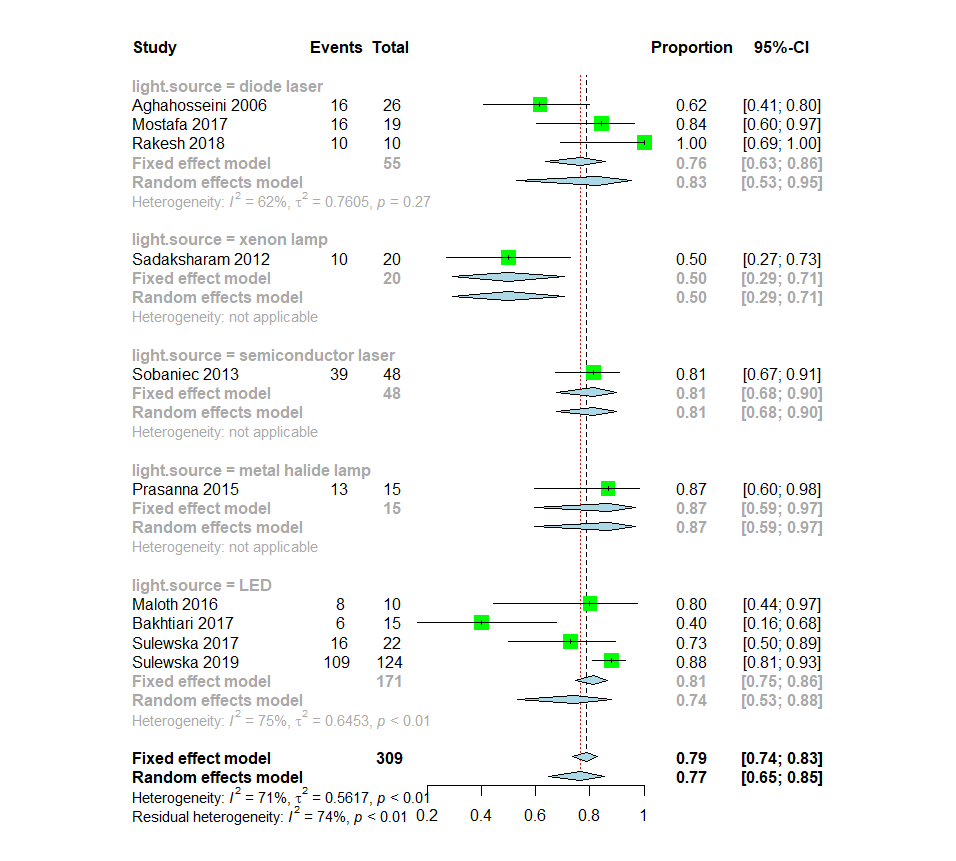


**Appendix Fig. 7** Forest plots of PR after PDT: subgroup analysis of light sources


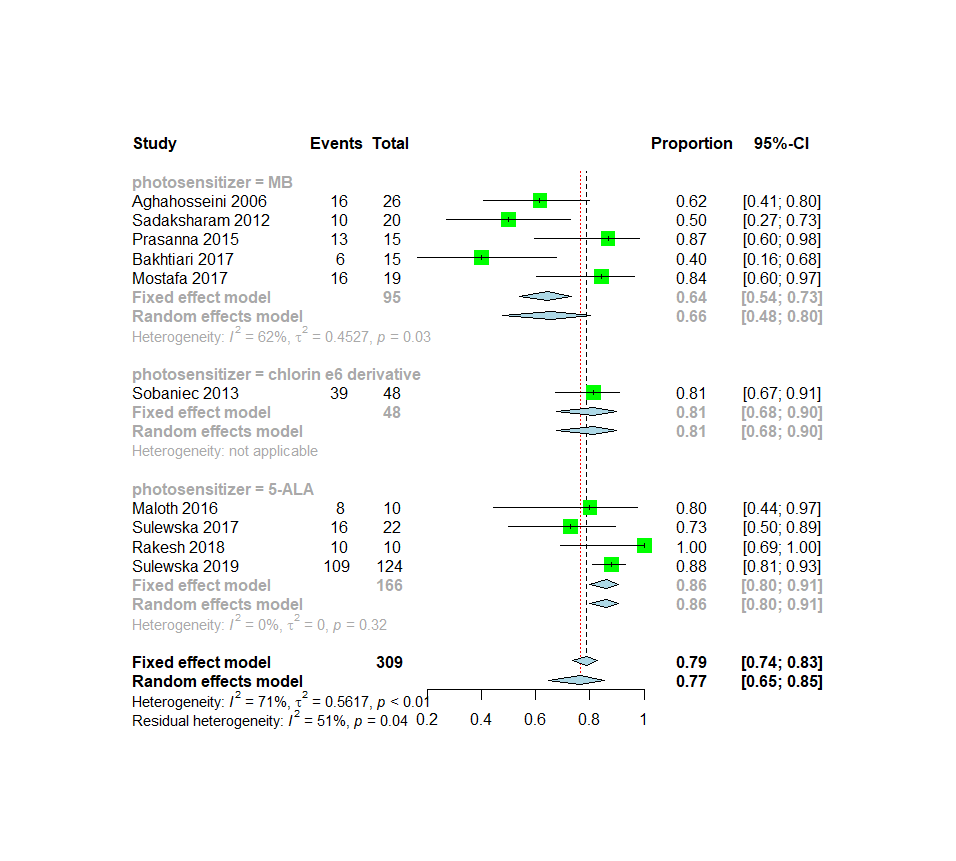


**Appendix Fig.8** Forest plots of PR after PDT: subgroup analysis of photosensitizers


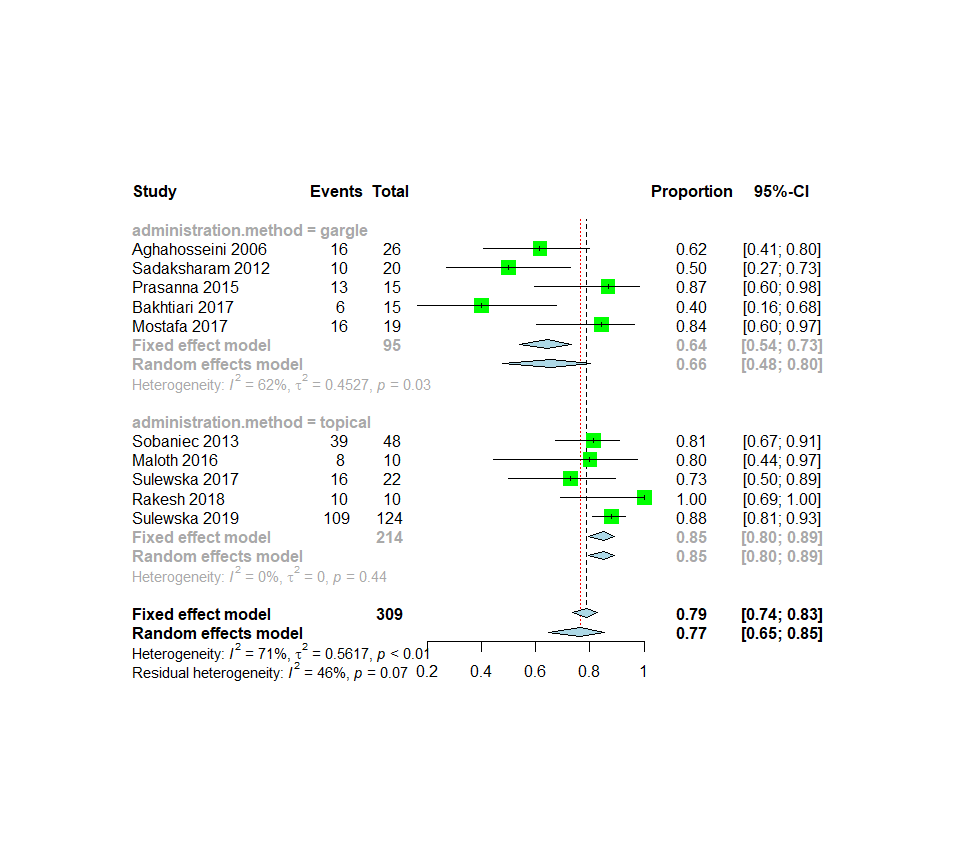


**Appendix Fig.9** Forest plots of PR after PDT: subgroup analysis of administration methods

**
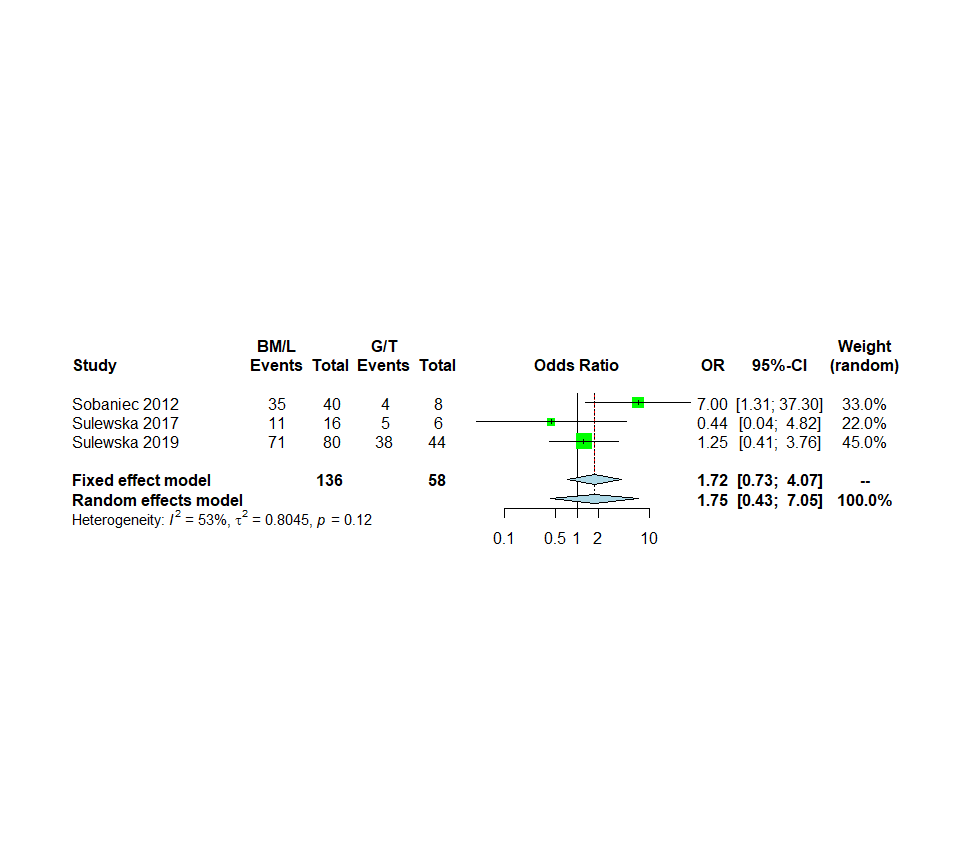
**

**Appendix Fig.10** Forest plot of PR after PDT: subgroup analysis of lesion locations

**
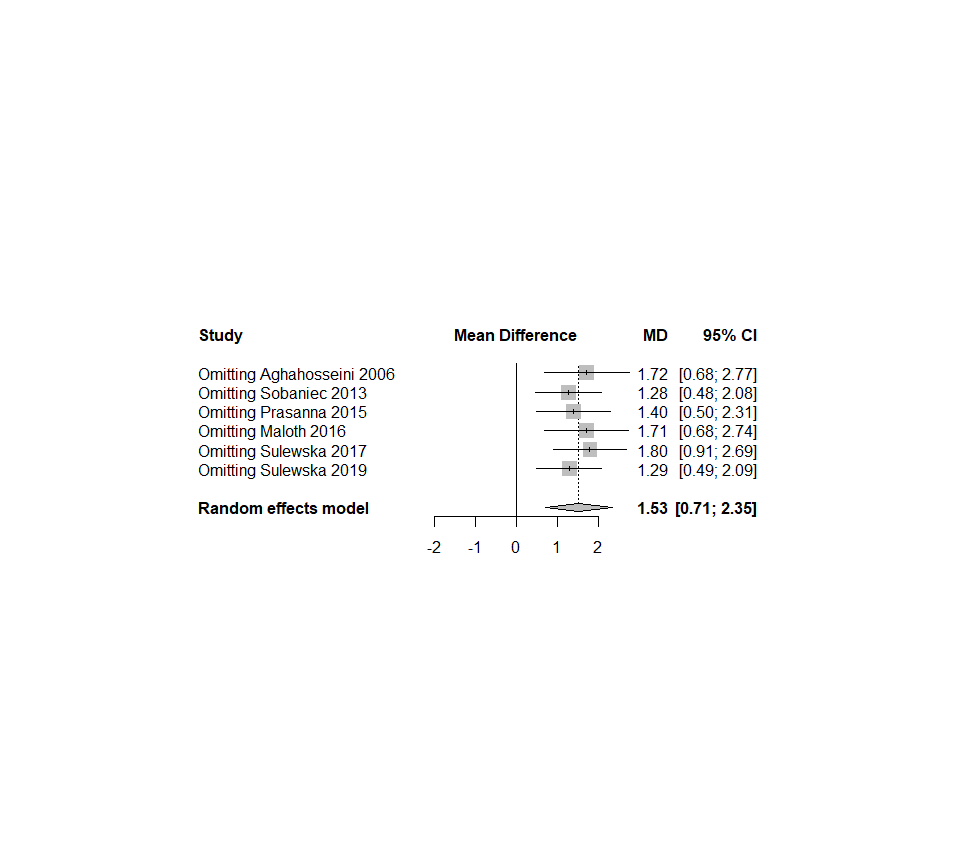
**

**Appendix Fig. 11** sensitivity analysis for stability of the changes of lesion size

**
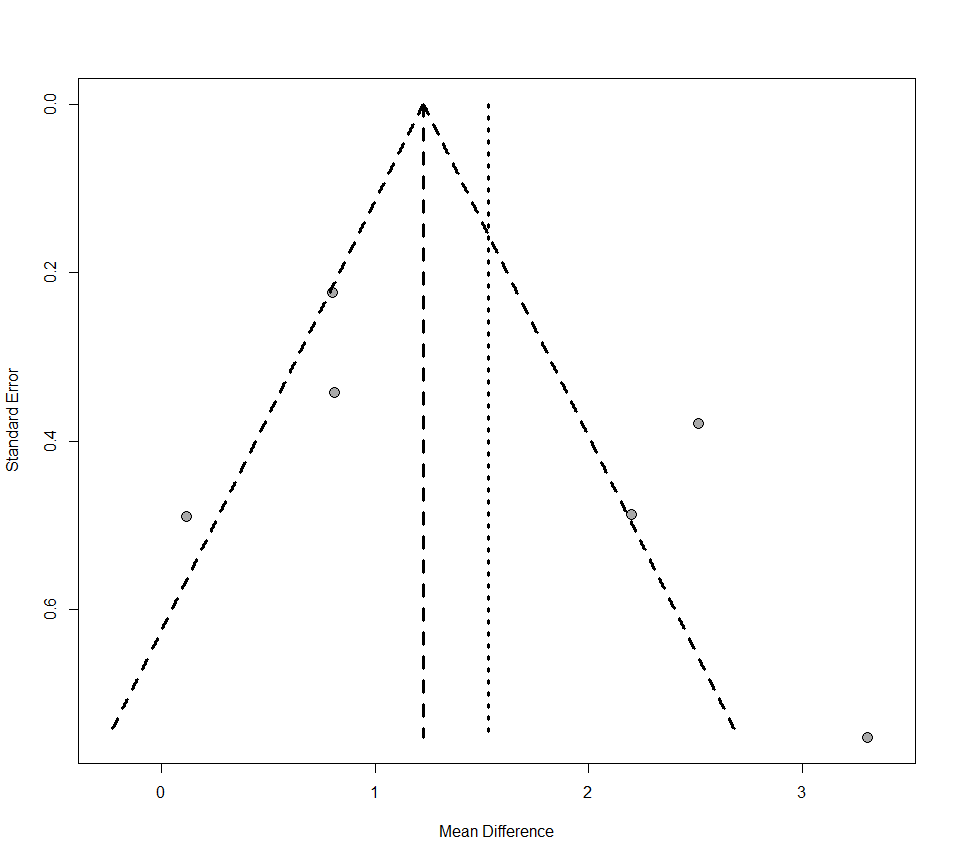
**

**Appendix Fig. 12** Funnel plot for lesion size after PDT

**
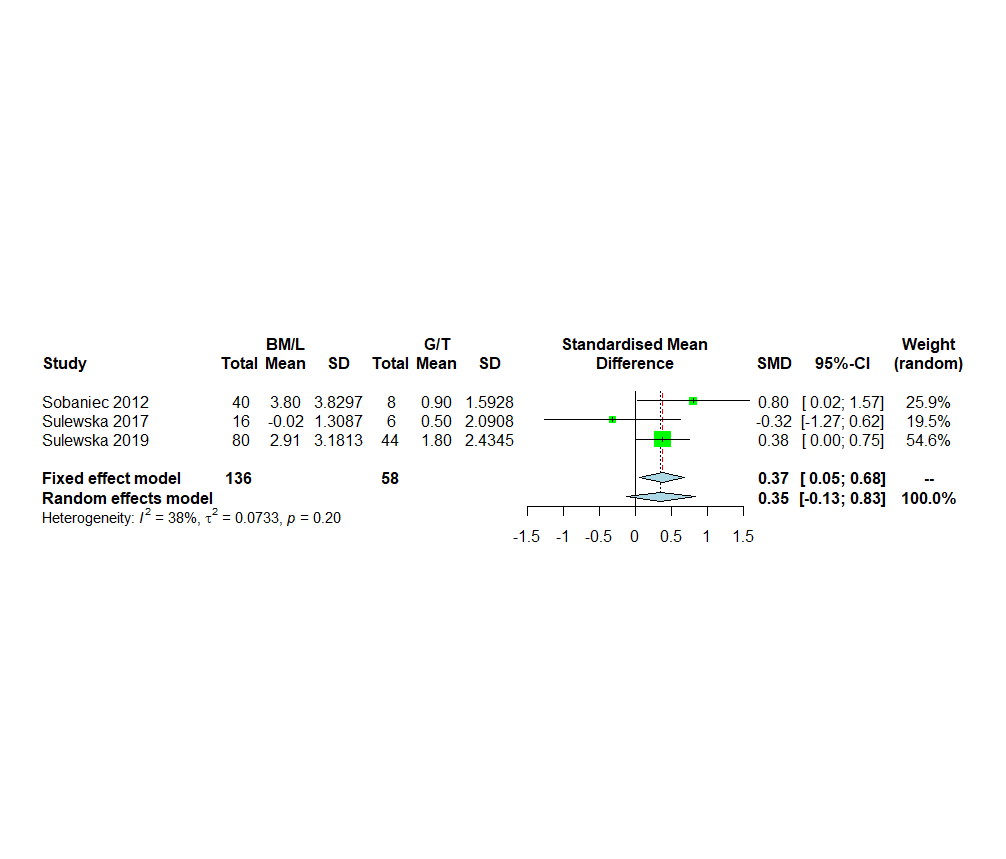
**

**Appendix Fig. 13** Forest plot of size after PDT: subgroup analysis of lesion location

**
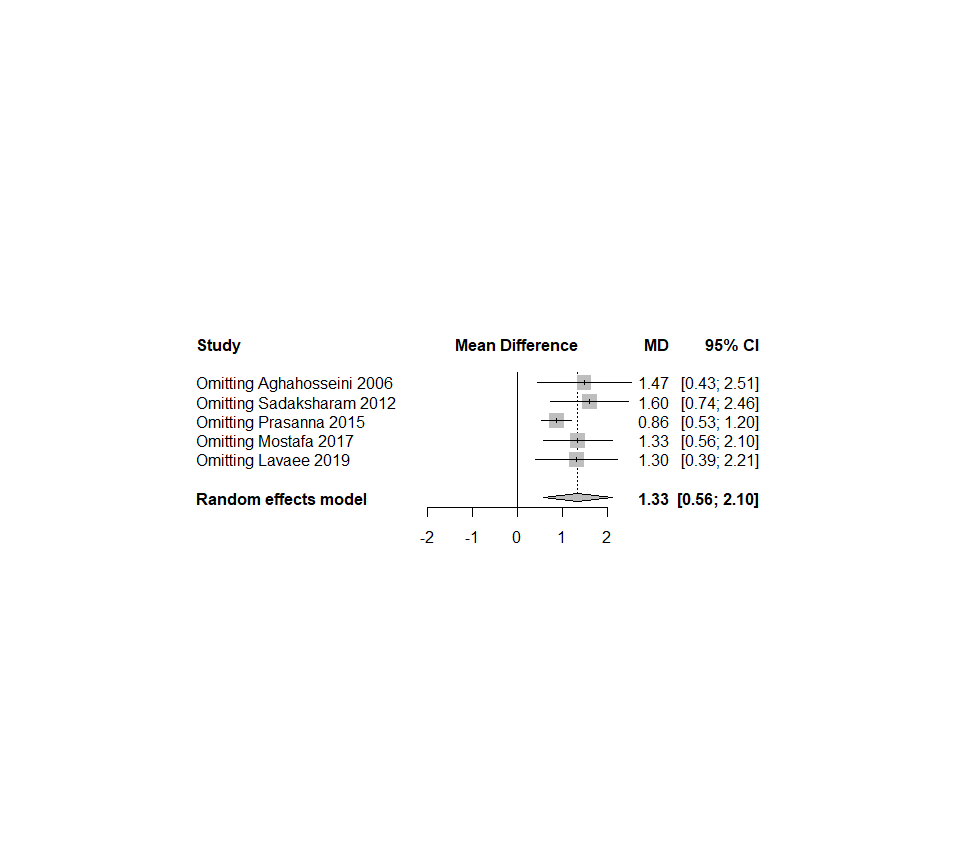
**

**Appendix Fig. 14** Sensitivity analysis for the results of TH

**
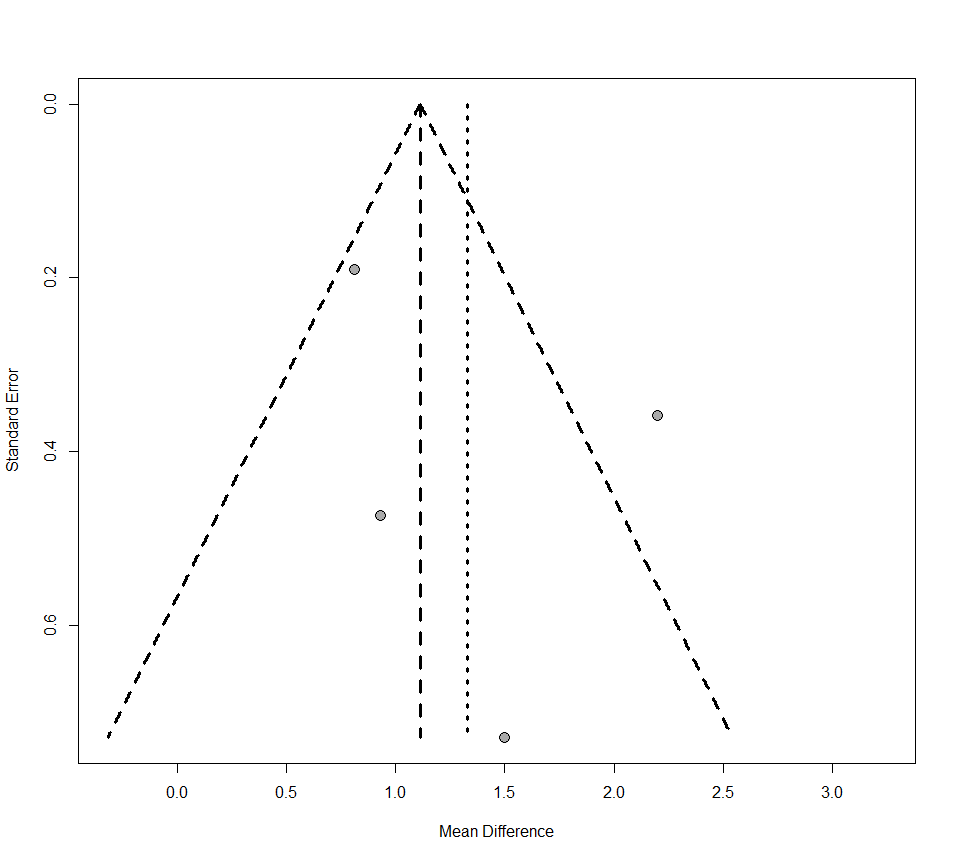
**

**Appendix Fig. 15** Funnel plot for TH after PDT.


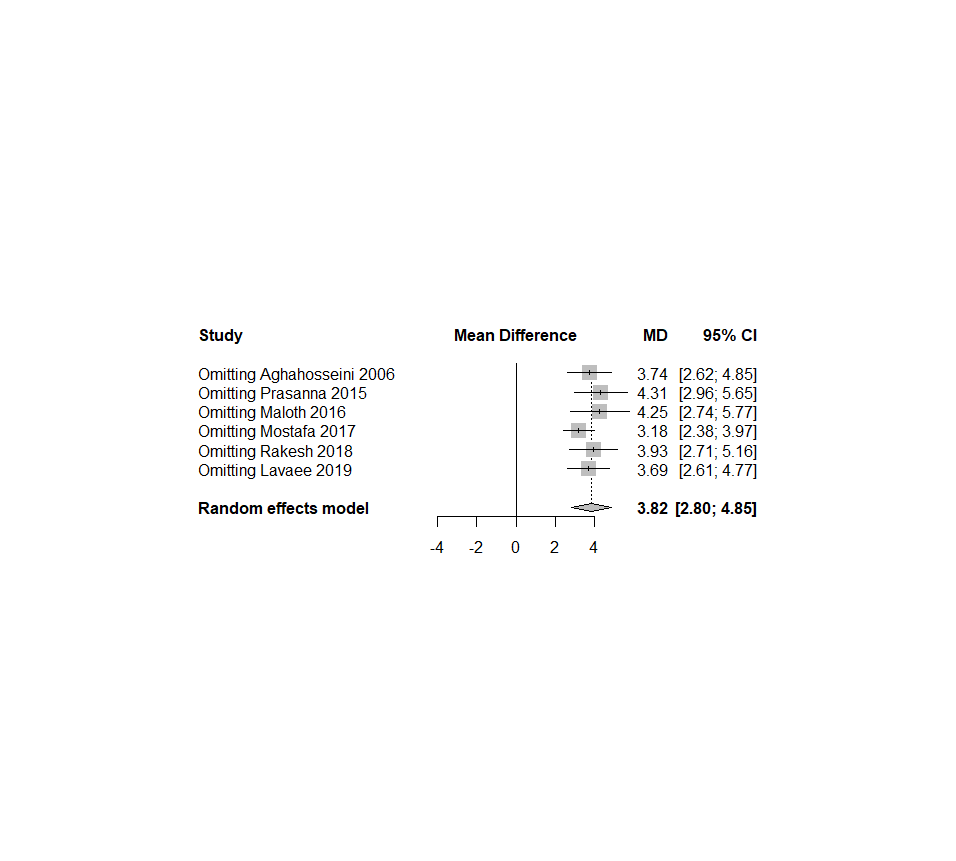


**Appendix Fig. 16** Sensitivity analysis for the results of VAS


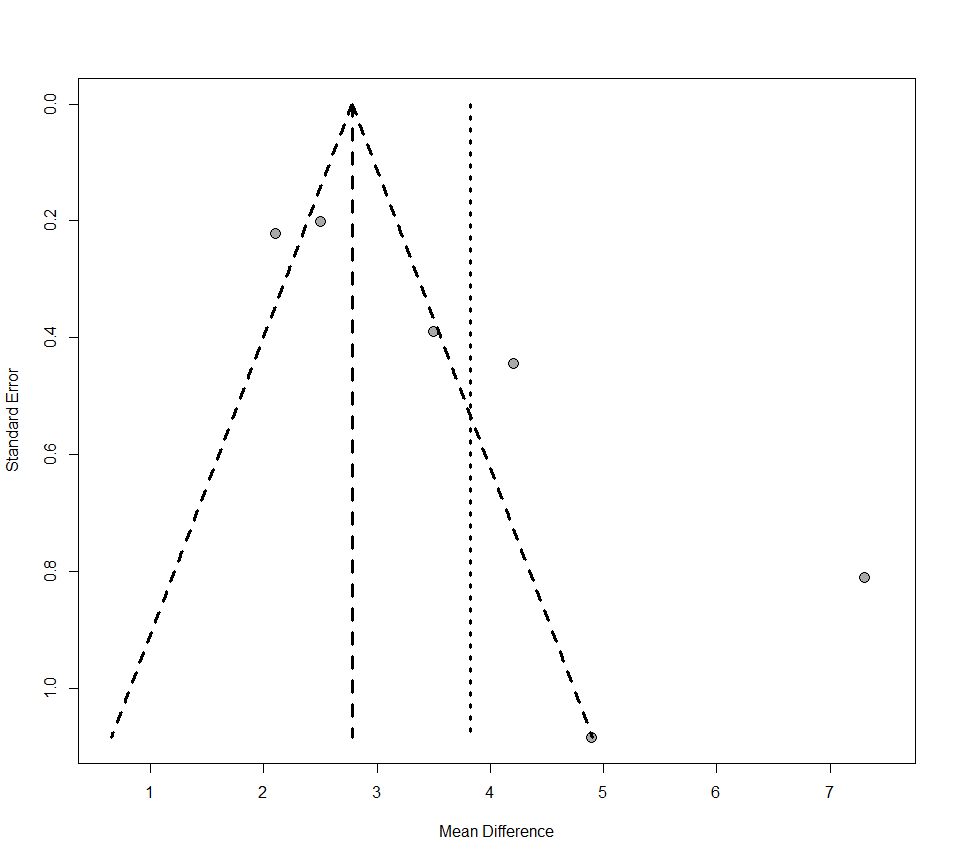


**Appendix Fig. 17** Funnel plot for VAS after PDT.

**
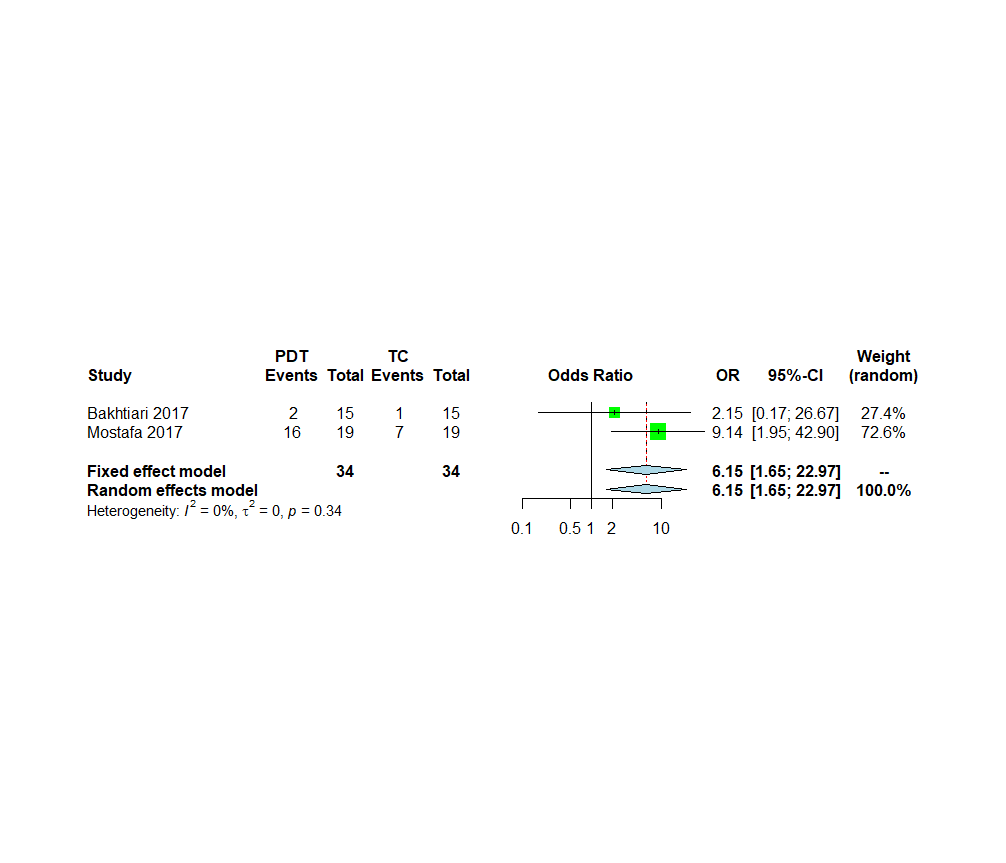
**

**Appendix Fig. 1****8** Forest plot of PDT comparing with topical corticosteroids on PR

**
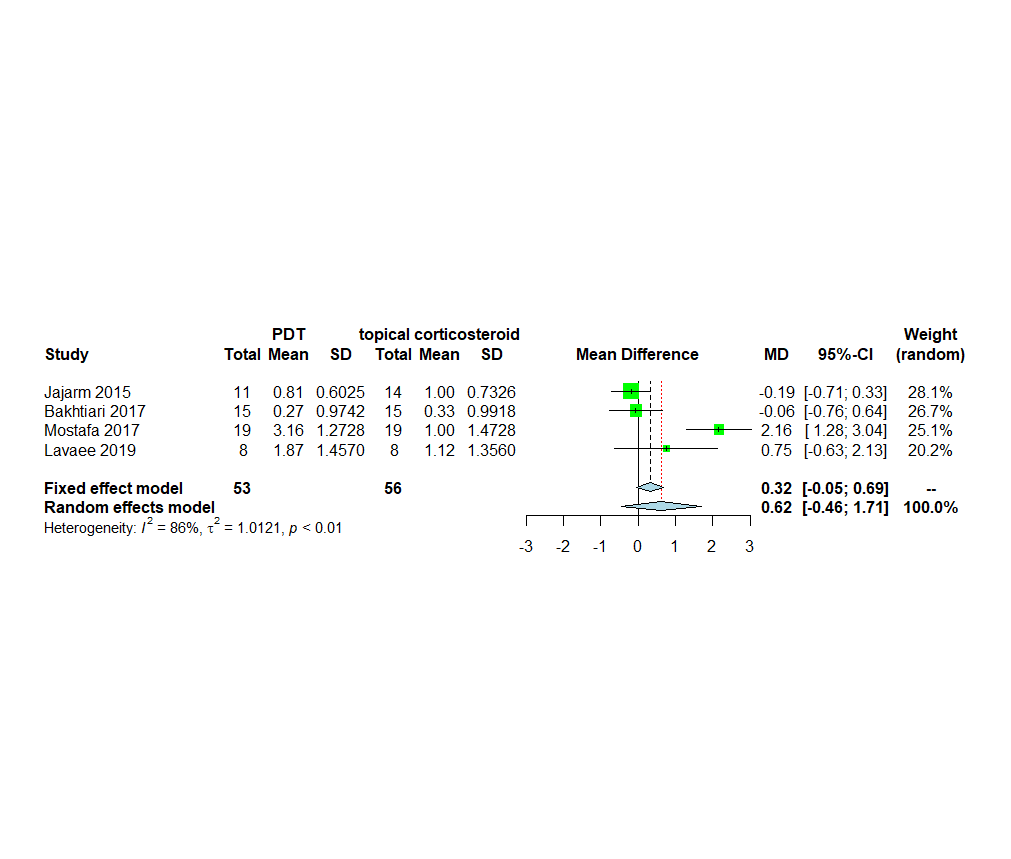
**

**Appendix Fig.19** Forest plot of PDT comparing with topical corticosteroids on TH

**
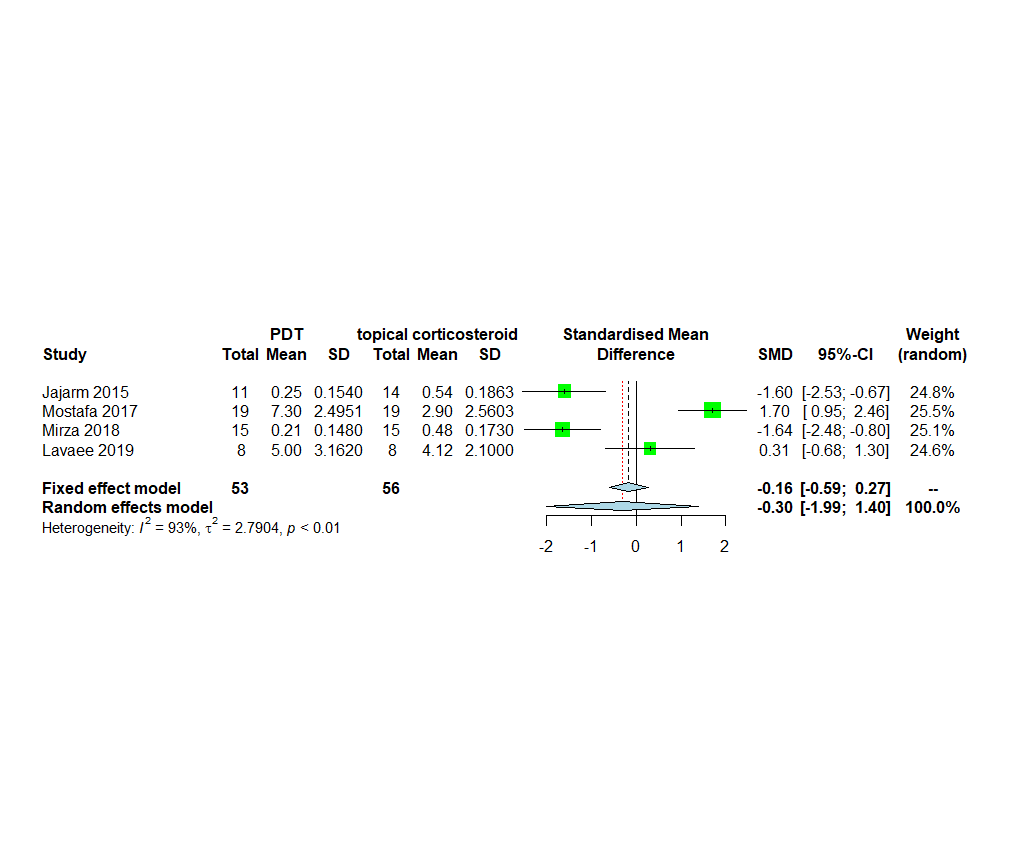
**

**Appendix Fig.20** Forest plot of PDT comparing with topical corticosteroids on VAS
